# Comparative Inter- and IntraSpecies Transcriptomics Revealed Key Differential Pathways Associated With Aluminium Stress Tolerance in Lentil

**DOI:** 10.3389/fpls.2021.693630

**Published:** 2021-08-31

**Authors:** Chandan Kumar Singh, Dharmendra Singh, Jyoti Taunk, Priya Chaudhary, Ram Sewak Singh Tomar, Shivani Chandra, Deepti Singh, Madan Pal, Noren Singh Konjengbam, M. Premjit Singh, Rakesh Singh Sengar, Ashutosh Sarker

**Affiliations:** ^1^Division of Genetics, Indian Agricultural Research Institute, New Delhi, India; ^2^Amity Institute of Biotechnology, Amity University, Noida, India; ^3^Division of Plant Physiology, Indian Agricultural Research Institute, New Delhi, India; ^4^College of Horticulture and Forestry, Rani Lakshmi Bai Central Agricultural University, Jhansi, India; ^5^Department of Botany, Meerut College, Meerut, India; ^6^College of Post Graduate Studies in Agricultural Sciences, Central Agricultural University—Imphal, Umiam, India; ^7^College of Agriculture, Central Agricultural University—Imphal, Iroisemba, India; ^8^College of Biotechnology, Sardar Vallabh Bhai Patel Agricultural University, Meerut, India; ^9^International Center for Agriculture Research in the Dry Areas, New Delhi, India

**Keywords:** aluminium stress, interspecific, intraspecific, lentil, transcriptomics, wild

## Abstract

Aluminium stress causes plant growth retardation and engenders productivity loss under acidic soil conditions. This study accentuates morpho-physiological and molecular bases of aluminium (Al) tolerance within and between wild (ILWL-15) and cultivated (L-4602 and BM-4) lentil species. Morpho-physiological studies revealed better cyto-morphology of tolerant genotypes over sensitive under Al^3+^ stress conditions. Mitotic lesions were observed in root cells under these conditions. Transcriptome analysis under Al^3+^ stress revealed 30,158 specifically up-regulated genes in different comparison groups showing contigs between 15,305 and 18,861 bp. In tolerant genotypes, top up-regulated differentially expressed genes (DEGs) were found to be involved in organic acid synthesis and exudation, production of antioxidants, callose synthesis, protein degradation, and phytohormone- and calcium-mediated signalling under stress conditions. DEGs associated with epigenetic regulation and Al^3+^ sequestration inside vacuole were specifically upregulated in wild and cultivars, respectively. Based on assembled unigenes, an average of 6,645.7 simple sequence repeats (SSRs) and 14,953.7 high-quality single nucleotide polymorphisms (SNPs) were spotted. By quantitative real-time polymerase chain reaction (qRT-PCR), 12 selected genes were validated. Gene ontology (GO) annotation revealed a total of 8,757 GO terms in three categories, *viz*., molecular, biological, and cellular processes. Kyoto Encyclopaedia of Genes and Genomes pathway scanning also revealed another probable pathway pertaining to metacaspase-1,−4, and −9 for programmed cell death under Al-stress conditions. This investigation reveals key inter- and intraspecies metabolic pathways associated with Al-stress tolerance in lentil species that can be utilised in designing future breeding programmes to improve lentil and related species towards Al^3+^ stress.

## Introduction

Lentil is a cold-season legume that belongs to the genus *Lens* and is considered to be the oldest cultivated legume, rich in proteins and fibre (FAOSTAT, 2016). Due to globally growing demands of lentil, its production has escalated from 4.9 million tonnes (Mt) to 6.3 Mt from 2010 to 2018 (FAOSTAT, 2019). Lentil cultivation areas in acidic soils are primarily affected by Al^3+^ toxicity, which affects plant growth and functioning during early stages; therefore, there is a dire need of addressing this major agricultural obstacle that will ultimately help in further production boost.

Plant sensitivity to Al^3+^ stress results in root stunting and death of lateral roots, even at micromolar concentrations of phytotoxic Al^3+^ ions. These ions also affect the cellular division process in the root tip meristem; which is immediately subdued under Al-stress conditions (Arunakumara et al., 2013). Toxic Al^3+^ ions cause chromosomal abnormalities such as bridges, chromosome stickiness, and reduced mitotic activities in root tips that leads to reduced root tip growth, resulting in brownish, stubby, and brittle roots (Zhang et al., 2014b; Li et al., 2015; Jaskowiak et al., 2018). Reduced root biomass results in diminished uptake of nutrients and water from the soil. Further, Al^3+^-induced phytotoxicity can induce lipid peroxidation in the root elongation zone together with reactive oxygen species (ROS) generation and callose accumulation in roots, etc. (Jaiswal et al., 2018). Callose is a polysaccharide deposited in roots in response to Al^3+^ toxicity and results in chelation of Al^3+^ ions, which further reduces the accumulation of Al^3+^ in roots (Yamamoto, 2019). Also, accumulated ROS are scavenged and detoxified through the production of osmolytes, organic acids, and antioxidant enzymes that protects plants under Al^3+^ toxic conditions (Pineros et al., 2005; Giannakoula et al., 2010). ROS detoxification through increased antioxidant enzyme activities of superoxide dismutase (SOD), peroxidases such as catalase (CAT), ascorbate peroxidise (APX), and guaiacol peroxidise (GPX), etc., has been reported in lentils under Al-stress conditions (Singh et al., 2016), which suggests the critical function of these enzymes in Al-resistance. Apart from that, Ryan et al. (2001) reported the exudation of various organic acids, such as oxalate, malate, and citrate, when roots were exposed to Al^3+^ ions, adding evidence to the fact that Al tolerance in plants can be improved by increased organic acid synthesis and exudation (Houde and Diallo, 2008).

It has been observed that mechanisms regulating Al-tolerance are different in various phyto-species under Al-stress conditions (Daspute et al., 2017). Besides, in some species, various mechanisms can work simultaneously, generating Al resilience through their combined effects. Although the type of tolerance-generation mechanisms for Al^3+^-induced phytotoxicity is controversial, as of now, Al exclusion mechanism is widely accepted to be involved in Al^3+^ detoxification (Arunakumara et al., 2013). Key genes coding for Al tolerance such as those involved in antioxidant enzyme activity; synthesis of organic acids such as pyruvate dehydrogenase kinase (PDK), isocitrate dehydrogenase, and succinate dehydrogenase; exudation of organic acids such as the Al-activated malate transporter (ALMT) and multidrug export protein AcrE (MATE) families, etc., have been identified in many crop plants (Furukawa et al., 2007; Saxena et al., 2011; De Angeli et al., 2013; Conde et al., 2007; Huang et al., 2017), but how they are regulated under Al-stress conditions in lentil cultivars and wild genotypes has not yet been studied. Thus, to develop Al-tolerant cultivars, it is imperative to have a deep understanding of different Al-tolerance mechanisms operating in lentils. Initially, efforts have been made to develop a comprehension of mechanisms for Al tolerance in lentil species. This unveiled Al exclusion mechanism as among the major mechanisms where organic acids or their anions were secreted from root apices that restricted the further attachment of toxic Al^3+^ ions to extra- and intra-cellular root components (Singh et al., 2016). Moreover, organic acid secretion was found maximum in wild genotypes (Singh et al., 2016). Apart from organic acid synthesis and exudation, ROS detoxification through antioxidant enzymes and callose deposition has also been reported in lentils (Singh et al., 2016).

The identification of key genes involved in regulatory mechanisms, metabolic pathways, and signalling pathways would facilitate a better understanding of Al tolerance mechanisms in lentils. To get a comprehensive view of regulatory mechanisms, RNA-seq has become a popular molecular technique especially for orphan crops or species with incomplete genome sequencing. It has been extensively employed to build draught transcriptomes in lentil (Sudheesh et al., 2016) and to unveil differentially expressed genes (DEGs) for drought stress (Singh et al., 2017), ascochyta blight (Khorramdelazad et al., 2018; Sari et al., 2018), cold stress (Barrios et al., 2017), and heat stress (Singh et al., 2019), etc.; but no investigation has been conducted in the case of Al stress so far. Information on genes involved in key regulatory pathways can be inferred from RNA-seq analysis and can be utilised for crop breeding programs. Therefore, this investigation was designed (i) to collate the morphological, physio-biochemical, and molecular rationale of Al tolerance between cultivated and wild lentil genotypes by genome wide transcriptome analysis, (ii) to elucidate Al-tolerance mechanisms in lentil genotypes, and (iii) to find key genes involved in Al tolerance regulation in wild and cultivated lentil species. The findings of this investigation will improve the strategies for lentil production under Al-toxicity conditions by targeting appropriate pathways responsible for Al-resistance or sensitivity by means of genetic engineering and/or targeted breeding, etc.

## Materials and Methods

### Plant Material

This investigation utilised three divergent genotypes of lentil, *viz*., BARI Masur-4 (BM-4), L-4602, and wild genotype ILWL-15. Genotypes BM-4 and L-4602 are categorised as under *Lens culinaris*, while ILWL-15 is classified as under *Lens nigricans*. Seeds of wild genotype (ILWL-15) were obtained from ICAR-National Bureau of Plant Genetic Resources (New Delhi, India). These lentil genotypes were selected for this study on the basis of the previous scrutiny (Singh et al., 2016), where the genotypes were categorised into contrasting groups for resilience to Al^3+^ stress. L-4602 is an Al-tolerant breeding line developed at Indian Council of Agricultural Research (ICAR)-Indian Agricultural Research Institute (IARI, New Delhi, India), while BM-4 is an Al-sensitive cultivar obtained by crossing genotypes ILL-5888, which is an improved landrace, with breeding line ILL-5782 at the International Centre for Agricultural Research in the Dry Areas, Syria (Gahoonia et al., 2005; Singh et al., 2016). ILWL-15 is a moderately tolerant, nutrient wealthy, and wild genotype that originated in France (Singh et al., 2016; Kumar et al., 2018).

### Growth Conditions and Al^3+^ Stress Treatment

Growth conditions as well as exposure to Al^3+^ stress of the lentil genotypes were provided and performed at National Phytotron Facility, ICAR-IARI (New Delhi, India). These were followed according to the methods described by Singh et al. (2016), with few modifications. By hydroponics, seedlings were grown in a growth chamber with temperature and humidity controls fixed at 27/16°C and 55.7/68.2 ± 2, respectively. The growth medium used for hydroponics contained 148 μM of AlCl_3_·6H_2_O (pH 4.8) for stress treatment; whereas, under control conditions, the medium was devoid of AlCl_3_·6H_2_O at pH 4.8. A completely randomised design with three replicates for each genotype was followed to expose the seedlings under both growth conditions.

### Morphological and Physio-Biochemical Variations Under Al^3+^ Stress Conditions

The relative root elongation (RRE) of the wild and cultivated genotypes was measured under both Al stress and control conditions at 3, 6, 12, and 24 h to determine its morphological impact. It is represented as percentage elongation of the root under Al^3+^ treatment conditions when compared with root elongation under control conditions (Al-free). Physio-biochemical changes in roots were deduced by estimating the Al^3+^ content, morin score, and callose score after 6 h of Al exposure, following the methods explained by Singh et al. (2016). Activities of antioxidant enzymes, *viz*., SOD, APX, and GPX along with proline were also estimated in the roots of cultivated and wild genotypes after 6 h of Al^3+^ exposure, following the methods of Singh et al. (2016). Estimation of total sugar and starch from the roots of contrasting genotypes under control and Al-treated conditions were performed following the method described by Mishra and Dubey (2008). Membrane stability index (MSI) was estimated following the method described by Chandra et al. (2020).

### Statistical Analysis

Morpho-physiological and biochemical traits were examined by two-way analysis of variance (ANOVA) to ascertain if the means were significantly different. Variances were inspected by plotting residual v/s fitted values to confirm the homogeneity of the data. Variations within the mean values were evaluated with least significant difference (LSD).

### Cytological Study for Changes in Chromosomal Structures

Seedlings were collected at 3, 5, and 24 h after aluminum^3+^ treatment to study changes in chromosomal structures in the root tips of lentil. Five root tips, each from control and treated seedlings, were taken and stained as described by Zhang et al. (2014a).

### RNA Isolation and Library Preparation

Total ribonucleic acid was isolated from the root samples (<1 cm) of sensitive and tolerant lentil genotypes under both control and Al-stress conditions using a Trizol reagent (Takara, Shiga, Japan). RNAs extracted from each biological replicate containing 12 seedlings were pooled together, and the test samples were labelled as “C” (for control) and “T” (for Al-treated) where numbers 1, 2, and 3 represent lentil genotypes, i.e., L-4602, BM-4, and ILWL-15, respectively. The extracted RNA was quantified using Nanodrop (Thermo Fisher Scientific, Waltham, MA, United States) and qualitatively determined by RNA integrity (RIN) test using Bioanalyzer (Agilent Technologies, Sta. Clara, CA, United States). Following the RNA quality assessment, a complementary DNA (cDNA) library was constructed using a Truseq RNA sample prep kit (Illumina, Inc., San Diego, CA, United States). Utilising a magnetic bead containing poly T-molecules, messenger RNA (mRNA) was fragmented, and selected RNA fragments were then converted to cDNA using Illumina TruSeq^TM^ mRNA Library Prep Kit (Illumina Inc., San Diego, CA, United States). For cluster generation, the ends of the selected fragments were ligated to paired end adapters (Illumina Inc. San Diego, CA, United States). cDNA enrichment was then performed by polymerase chain reaction (PCR) amplification of fragments having adapters ligated to both of their ends. The cDNA library was then sequenced using an Illumina HiSeq 2500 platform (Illumina Inc., San Diego, CA, United States). Using TruSeq RNA fragmentation protocol, cDNA fragments of 120–200 bp with a median of 150 bp were generated. Fragments from each sample were eluted according to bead volume and incubation time at consecutive steps. Thereafter, 100 bp reads were selected from each sample that generated total raw reads from 19,500,000 to 32,185,339.

### Contig Assembly and Differential Expression of Genes

Raw Fastq files were filtered for any low-quality reads [Phred quality (Q) score < 20] using FastXToolkit (Version 0.0.13). The FASTQC tool (Version 0.11.8) was used to assess the quality of reads pre- and post-filtering of raw reads. Trinity assembler version v2.12.0 was used to assemble the filtered reads with good quality to obtain *de novo* contigs. Non-extendable unigenes or non-redundant transcripts that qualified redundancy exclusion were identified with the help of sequence clustering software. The Bowtie (Version 1.2.2) program was utilised to map clean reads to assembled transcripts. Using the Edge R package, an analysis of differential gene expression was performed. To avoid the inflation of type-1 errors, significant DEGs based on false detection rate (FDR) value <.05 in various comparison groups, *viz*., 1T v/s 2T v/s 3T, 1C v/s 1T, 2C v/s 2T, and 3C v/s 3T, were determined. Significant DEGs were assorted on the basis of descending absolute log_2_fold change (logFC). The contrast between contigs of the different comparison groups was depicted using circus plots. Heat maps were used to represent top significant DEGs with logFC > 4 for the different comparison groups.

### Gene Ontology Enrichment Analysis

To determine the unigene identity, the National Centre for Biotechnology Information (NCBI)- basic local alignment search tool (BLAST) was used with fixed attributes of sequence similarity index up to an E-value < 1 E^−5^ using various public reference databases such as UniProt Reference Clusters (UNIREF), NCBI non-redundant (nr), and Swiss-Prot. Unigene classification was performed using the Annocript program (version 2.0.1). DEGs were characterised into three major functional categories, *viz*., cellular components, molecular functions, and biological processes. To make GO plots for filtered transcripts, Web Gene Ontology Annotation Plot (WEGO) was utilised. The Shinygo database was used to deduce the GO term enrichment scrutiny of topmost 50 DEGs. Cataloguing of unigenes was performed using the Cluster of Orthologous Groups (COG) database. Corresponding metabolic pathways were established through the Kyoto Encyclopedia of Genes and Genomes (KEGG) database. DEGs obtained from various contrasting groups were represented by different plots, e.g., Circos plots, Volcano plots, HeatMaps, and MapMan (version 3.51R2).

### Quantitative Real-Time PCR-Based Validation of DEGs and Their Functional Correlation

The total RNA extracted previously for cDNA library preparation was used for validation of DEGs following qRT-PCR. The cDNA was manufactured using a verso cDNA kit (Thermo Fisher Scientific, Waltham, MA, United States). The cDNA was amplified with One Step SYBR PrimeScript RT-PCR Kit II (Takara Biotechnology Co. Ltd, Dalian, Japan). Four biological replicates for each sample were followed. Total 100 ng cDNA was added in a 10-μl PCR mixture. Using the 2^−(ΔΔCT)^ method, the differential gene expression of targeted genes was calculated and calibrated in CFX96 RT-PCR (Bio-Rad, Berkeley, CA, United States). A PCR reaction mixture (25 μl) composed of template cDNA of 6.8 μl, 2x SYBR Green mix of 10 μl, and a total of 10 μl primers (Integrated DNA Technologies, San Diego, CA, United States) was used for validation. To normalise the expression data of target genes, housekeeping gene β-tubulin was taken. Primers utilised for qRT-PCR validation are designed with the Primer3Plus program (https://primer3plus.com/cgi-bin/dev/primer3plus.cgi) and are represented in a Supplementary Table 1. DEGs were functionally correlated to corresponding proteins *via* a proteomics study. The latter was performed to detect the abundance of cell wall biogenesis and modification proteins in roots of the Al-tolerant and sensitive genotypes. Protein extraction, trypsinisation, and cleaning up were performed from the roots of tolerant and sensitive cultivars exposed to control and Al-treated conditions following the protocol described by Isaacson et al. (2006). Liquid chromatography with tandem mass spectrometry (LC-MS/MS) was performed using EASY-nLC 1200 UPLC and a Thermo Scientific Q Exactive plus hybrid quadrupole-Orbitrap mass spectrometer system (Thermo Fisher Scientific, Waltham, MA, United States) for all the samples, following the protocol described by Han et al. (2019). The MS/MS data generated from the LC-MS/MS profiling of peptides were then processed using Proteome Discoverer with an integrated SequestHT search engine. Tandem mass spectra were searched against the Uniprot *Glycine max* and *Medicago truncatula* database. The mass error was set to 10 ppm for precursor ions and.02 Da for fragment ions.

### Variant Calling and Filtering

Markers such as SNPs and SSRs were called using the Samtools mpileup and MIcro SAtellite identification (MISA) software, respectively. Twenty base pairs of read depth were selected to sort out false positive SNPs and heterozygous loci. Furthermore, SNPs were then identified using Genome Analysis Tool Kit (GATK) version 3.6-0. Using MISA, SSRs present in top-quality filtered reads were discovered. The primer design of SSRs was created using the Primer3 software. Data input settings were adjusted to a lowest primer length of 15 bp and highest of 21 bp with an optimal length of 18 bp. The range of product was fixed to 100–300 bp.

## Results

### Phenotyping of Al Stress Tolerance Based on Root Elongation Rate

**Figure 1:**
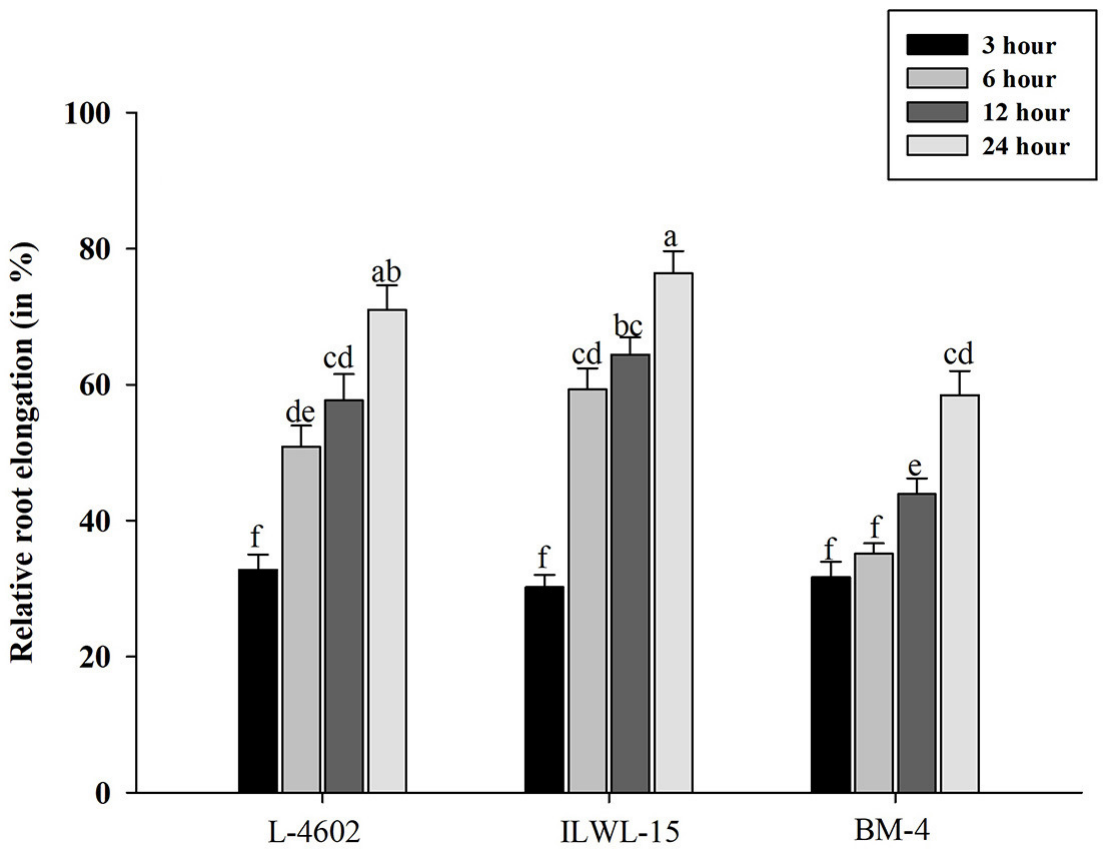
Relative root elongation (%) of aluminium (Al)-tolerant (L-4602, ILWL-15) and sensitive (BM-4) lentil genotypes at 3, 6, 12, and 24 h of Al-treatment (148 μM AlCl_3_.H_2_O). Mean values (*n* = 3) amid same small letters (a, b, c, d) are not statistically different. Error bars represent ± standard error (SE).

Tolerant and sensitive genotypes were assessed at 3, 6, 12, and 24 h for RRE after exposure to 148 μM Al^3+^ (Figure 1). The genotypes varied in their response towards Al^3+^ stress with increase in time period. The tolerant genotypes showed maximum RRE as compared with the sensitive ones in response to Al stress. Higher RRE denoted lesser damage to root under Al^3+^ stress conditions. A prominent difference was noticed after 6 h of Al treatment with RRE of 50.87, 59.3, and 35.13% in the L-4602, ILWL-15, and BM-4 genotypes, respectively. Therefore, Al treatment at 6 h was considered appropriate for evaluating physio-biochemical, genetic, and molecular parameters.

### Physio-Biochemical Components of Al-Stress Tolerance

**Figure 2:**
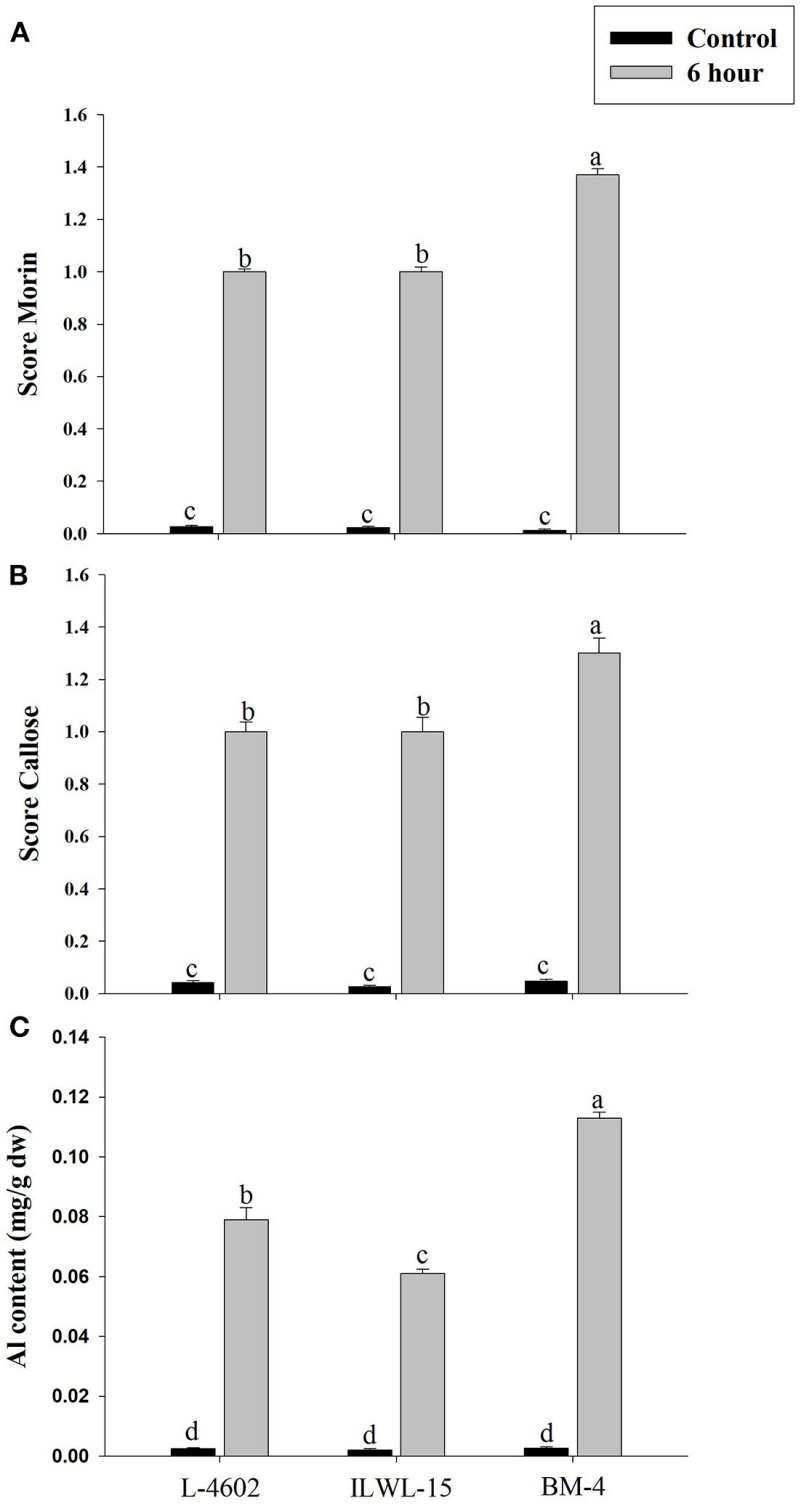
Differential aluminium stress response in roots of tolerant (L-4602, ILWL-15) and sensitive (BM-4) lentil genotypes. **(A)** Level of aluminium accumulation depicted through morin score; **(B)** callose deposition depicted through aniline blue staining; and **(C)** Al content in roots. Mean values (*n* = 3) amid same small letters (a, b, c, d) are not statistically different. Error bars represent ± SE.

**Figure 3:**
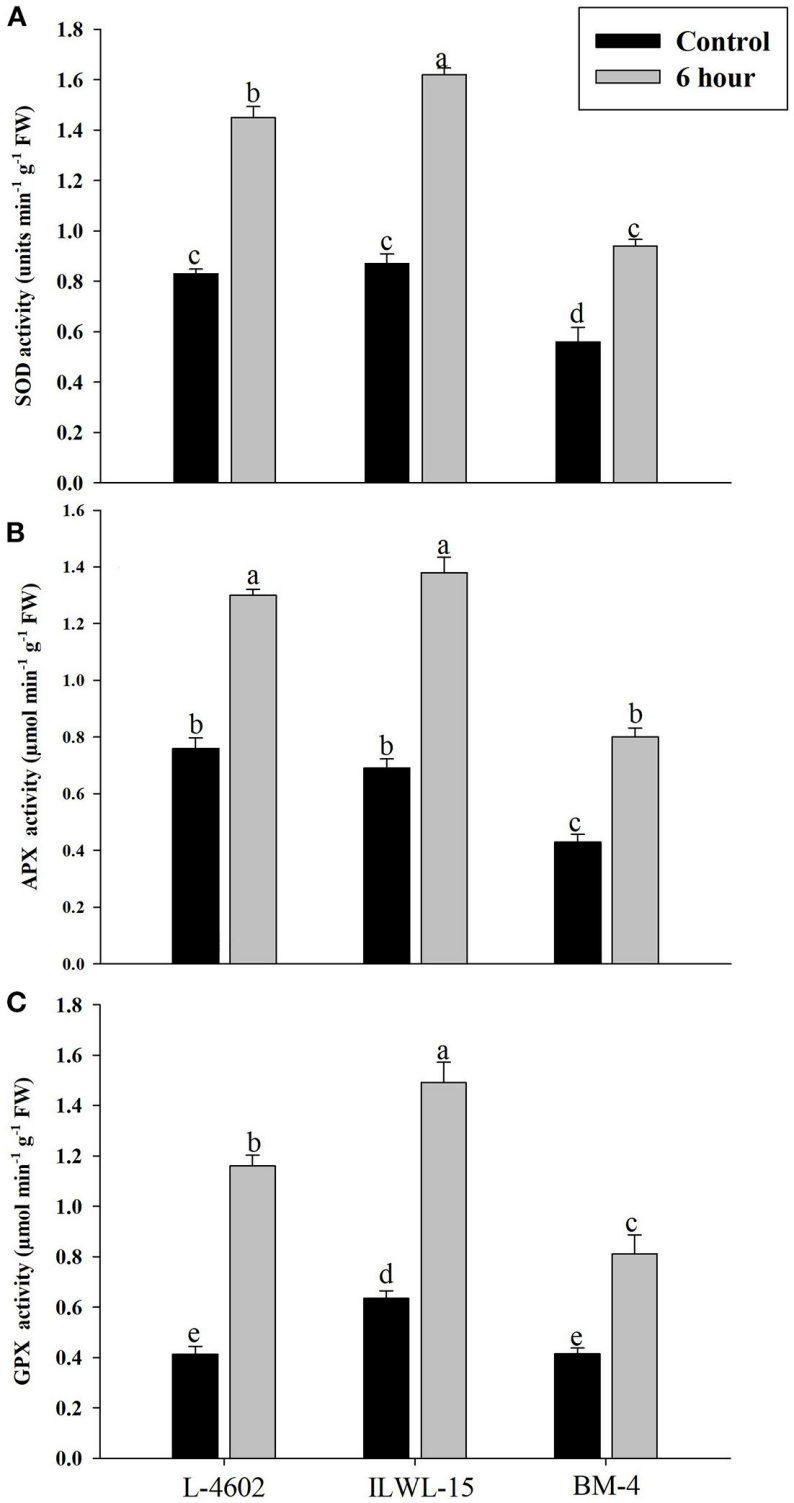
Response of antioxidant enzymes **(A)** superoxide dismutase (SOD); **(B)** ascorbate peroxidise (APX); and **(C)** guaiacol peroxidise (GPX) in roots of aluminium-tolerant (L-4602, ILWL-15) and sensitive (BM-4) lentil genotypes under control and treated (148 μM AlCl_3_·H_2_O) conditions. Mean values (*n* = 3) amid same small letters (a, b, c, d) are not statistically different. Error bars represent ± SE.

**Figure 4:**
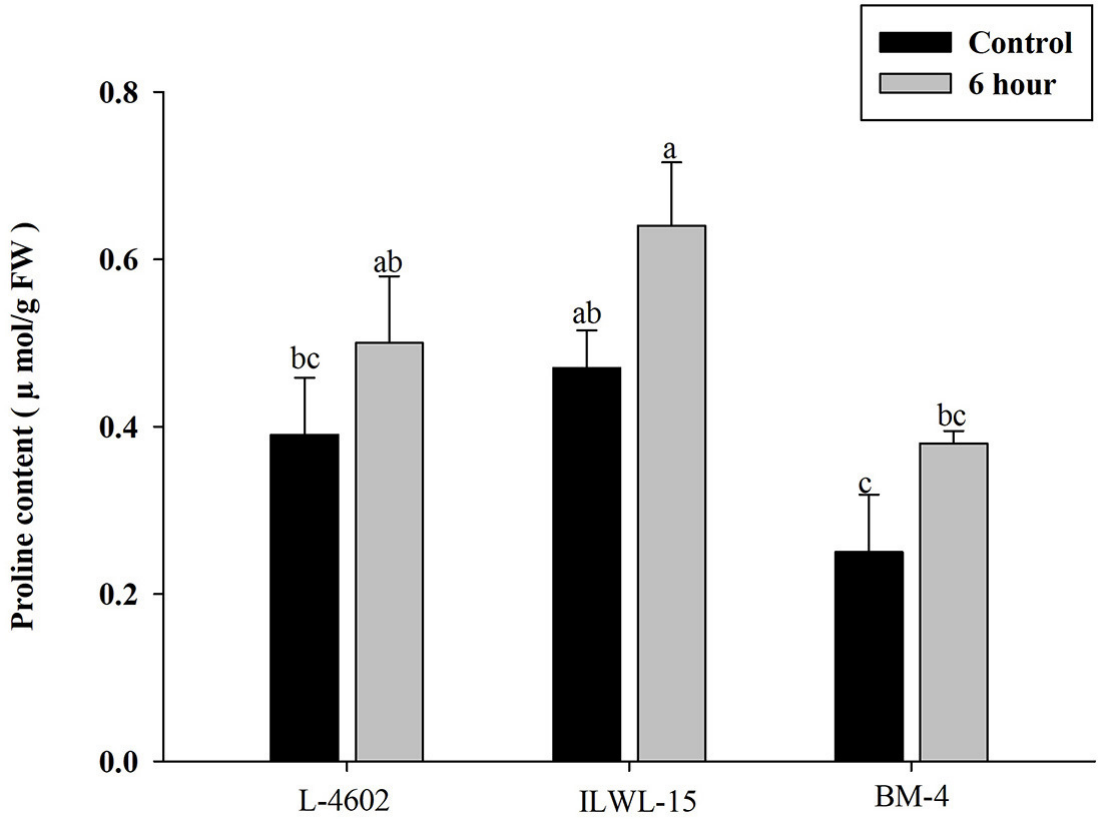
Accumulation of proline in roots of aluminium tolerant (L-4602, ILWL-15) and sensitive (BM-4) lentil genotypes under control and treated (148 μM AlCl_3_.H_2_O) conditions. Mean values (*n* = 3) amid same small letters (a, b, c, d) are not statistically different. Error bars represent ± SE.

Both Al and callose accumulation in roots increased after 6 h of exposure to 148 μM Al^3+^. Lower Al content was detected in root tips of the Al-tolerant genotypes (L-4602, ILWL-15) than in the Al-sensitive (BM-4) genotype. The tolerant genotypes showed less fluorescence signals when morin staining was performed, and indicated lower accumulation of Al^3+^ than the sensitive genotype (BM-4). The difference in Al^3+^ tolerance between the contrasting genotypes was also determined based on callose accumulation in reaction to Al^3+^ stress. Al-tolerant genotypes L-4602 and ILWL-15 visualised lesser callose in root tips than Al-sensitive BM-4 in response to Al^3+^ stress (Figure 2). Activities of antioxidant enzymes, such as SOD, APX, GPX, and proline content were increased in roots at 6 h under Al^3+^ stress conditions than control conditions in all the genotypes (Figure 3). Increase in antioxidant activities was maximum in the Al-tolerant genotypes than the Al-sensitive one (Figures 3A–C). Accumulation of proline was also induced under Al^3+^ stress conditions in all the genotypes (Figure 4). However, it tends to be higher in the tolerant genotypes and the wild genotype than the sensitive genotype.

**Figure 5:**
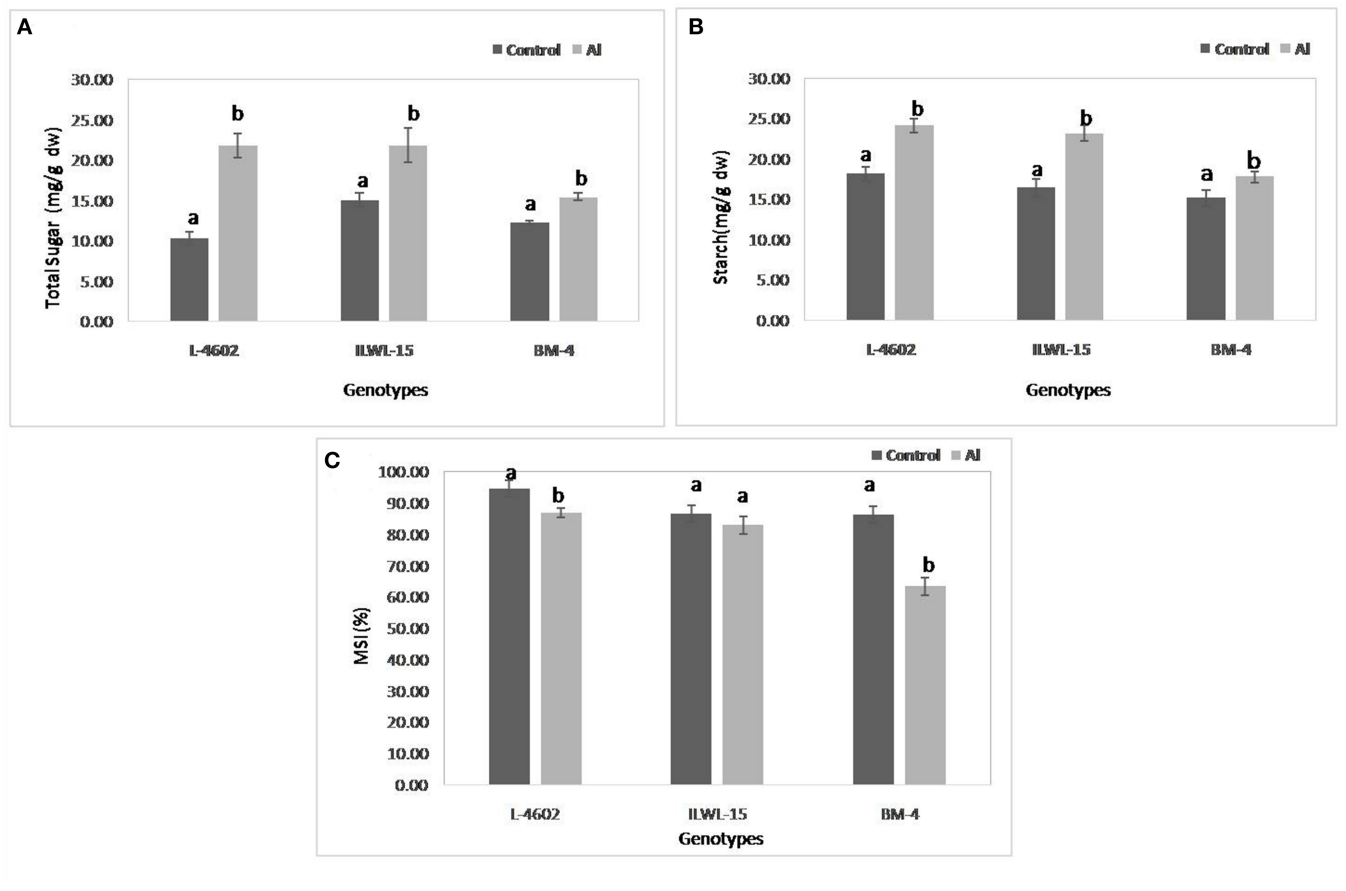
Changes in **(A)** total sugar; **(B)** starch; and **(C)** membrane stability index (MSI) in aluminium tolerant (L-4602, ILWL-15) and sensitive (BM-4) lentil genotypes under control and treated (148 μM AlCl_3_·H_2_O) conditions. Mean values (*n* = 3) amid same small letters (a, b, c, d) are not statistically different. Error bars represent ± SE.

Both total sugar and starch contents were also increased significantly following 6 h of Al-exposure, where the increase was higher in the tolerant cultivars and the wild genotype as compared with the sensitive one (Figures 5A,B). MSI was reduced under Al-stress conditions when compared with control conditions, although highly significant reductions were observed in the case of the sensitive cultivar (22.9%) as compared with the tolerant ones (7.7%). The wild genotype did not exhibit any significant reduction in MSI after Al-treatment (Figure 5C).

### Mitotic Changes Due to Al^3+^ Toxicity

**Figure 6:**
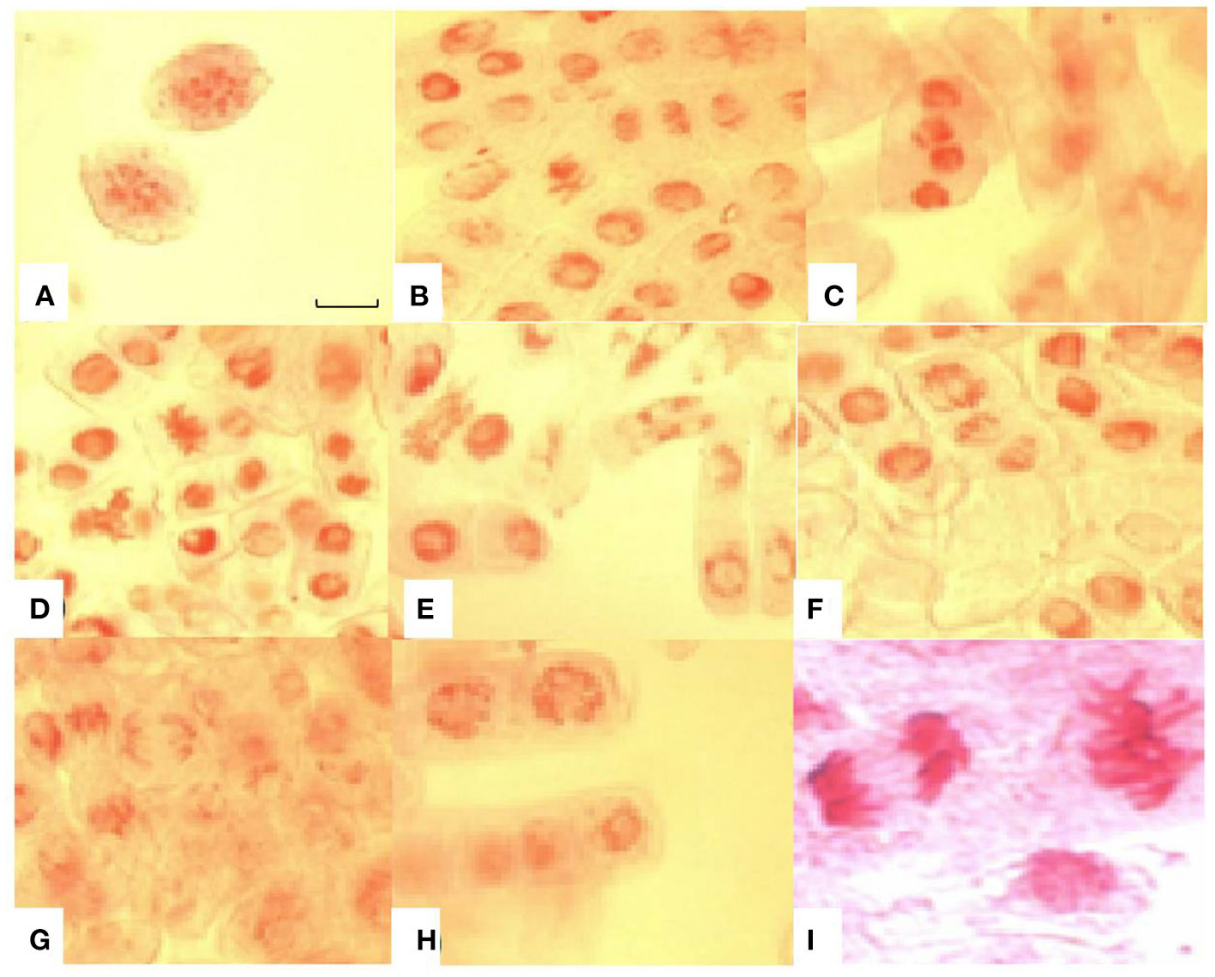
Chromosomal aberrations induced by aluminium toxicity in root tip cells of sensitive lentil genotype (BM-4). **(A)** chromosome fragments; **(B)** sticky chromosomes at metaphase and anaphase stages; **(C)** coagulated anaphase stage; **(D)** sticky chromosome at metaphase stage; **(E)** chromosomal bridges; **(F)** broken chromosomal bridges at metaphase and sticky chromosomes at anaphase; **(G)** broken bridges at anaphase; **(H)** nuclear lesions; **(I)** sticky chromosomes at anaphase and metaphase. Bar in figure represents 100 μm.

Various types of chromosomal abnormalities such as chromosome fragmentation, stickiness, bridges, and coagulation were also observed in this study. Apart from chromosomal aberrations, nuclear lesions were a common abnormality in root tip cells with increased time of exposure to Al^3+^ stress. Chromosomal bridges and coagulation at anaphase stage were amongst the significant aberrations found in lentil under Al^3+^ stress (Figure 6). Chromosome bridges can break and give rise to chromosomal fragments, which are clearly visible in this study.

### RNA Quality Assessment and Selection of Reads

The RNA integrity number (RIN) of each sample was in the satisfactory range of 6.7–7.9 under both control and Al-stress conditions. Clean, filtered reads obtained from TruSeq fragmentation were assembled, and contigs were found to range between 15,305 and 18,861 bp. Lowest contig length was set at 500 bp, whereas the highest average length after the *de novo* assembly of reads was found to be 18,361.7 bp for sample 2T under Al-treatment conditions. The average number of contigs N_50_ ranged from 1,051.6 to 1,810.06 for the different samples. The average reads of each sample depicting major characteristics of annotation and assembly are presented in Table 1.

**Table 1:** Transcriptome profile of tolerant and sensitive genotypes under control and aluminium stress conditions.

	**CT (Control)Average**			**Al (Treated) Average**		
	**1C**	**2C**	**3C**	**1T**	**2T**	**3T**
Total number of contigs	45,461	48,727.6	28,399	40,862.5	44,735	37,998.8
Maximum length of contigs	15,999.3	15,223.7	15,305	15,509.3	18,361.7	163,92
Minimum length of contigs	500	500	500	500	500	500
Average contigs length	1,448.1	1,464.9	991.6	1,301.5	1,469.8	1,254.3

*1C: L-4602 control, 2C: BM-4 control, 3C: ILWL-15 control, 1T: L-4602 Treated, 2T: BM-4 Treated, 3T: ILWL-15 Treated*.

### Differential Expression of Genes

**Figure 7:**
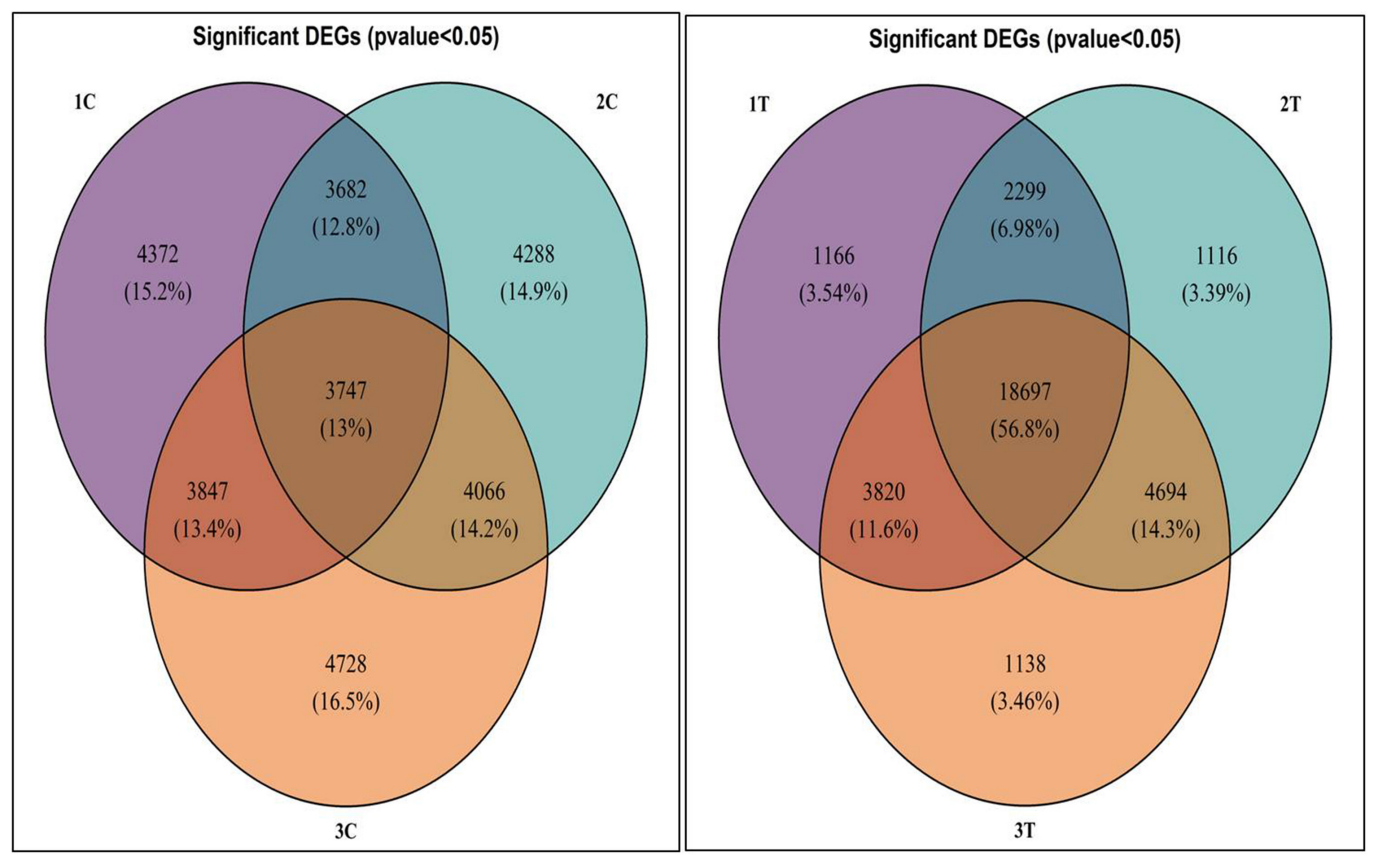
Venn diagram representing significant differentially expressed genes (DEGs) following Al stress (148μM AlCl_3_·H_2_O) in lentil genotypes in different comparison groups where 1C: L-4602 control, 2C: BM-4 control, 3C: ILWL-15 control, 1T: L-4602 treated, 2T: BM-4 treated, and 3T: ILWL-15 treated.

**Figure 8:**
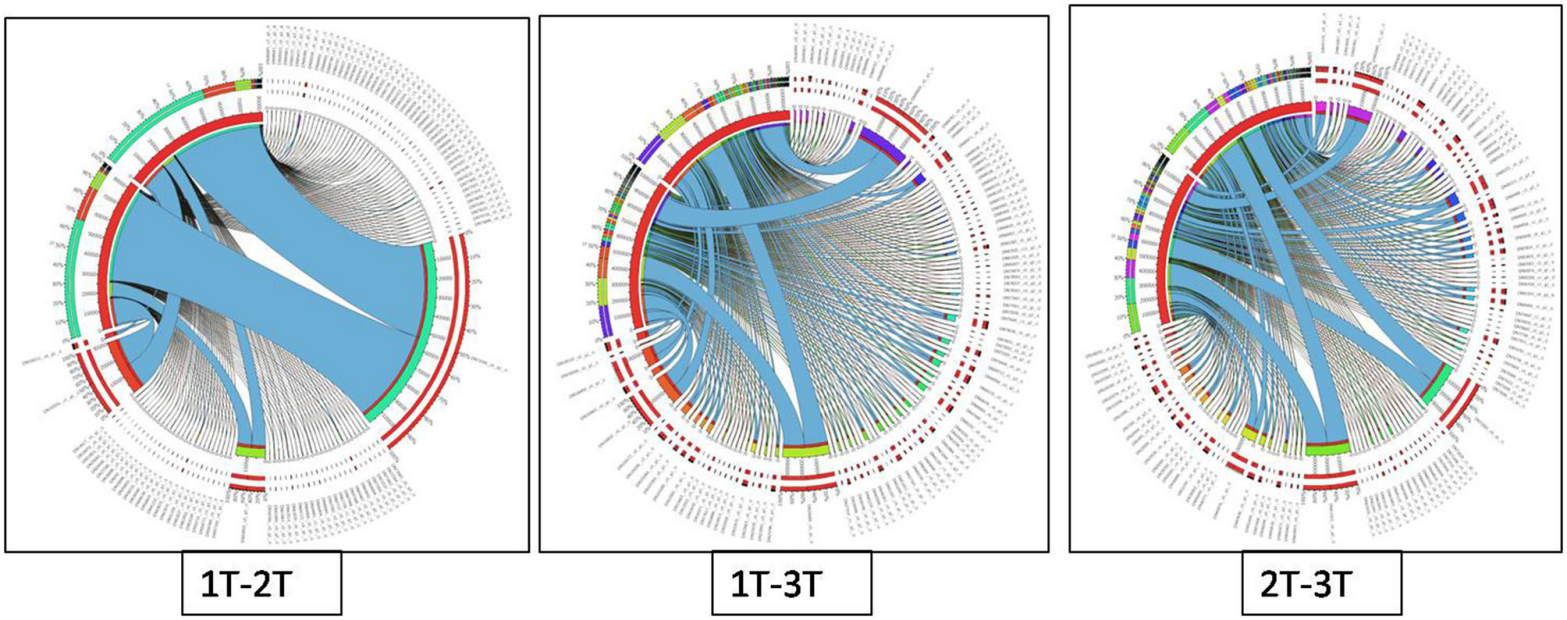
Circos plot representing the distribution of contigs in lentil genotypes following Al stress (148 μM AlCl_3_·H_2_O) for combinations 1T-2T, 1T-3T, and 2T-3T where, 1T: L-4602 treated, 2T: BM-4 treated, and 3T: ILWL-15 treated. Ribbons represent rows and column represents IDs. The three outer rings are stacked bar plots that represent the relative contribution of a cell to row and column totals. Expression value is expressed in colours, with red representing lowest value and purple representing highest values.

Differentially expressed genes were considered significant and showed *Q*-value/FDR/adjusted *p* < 0.05 and Log_2_FC changes > 1.5 for the different comparison groups (Supplementary Table 2). The Edge R program software deduced a total sum of 39,515 up-regulated transcripts and 37,890 down-regulated transcripts in the contrasting groups. Using Venn diagrams, shared significant contigs within various comparison groups along with discrete contigs are represented in Figure 7. Comparing the tolerant, sensitive, and wild genotypes with their respective controls, a total of 2,329, 2,380 and 5,130 up-regulated DEGs, and 1,609, 1,562, and 2,420 down-regulated DEGs were observed, respectively. The distribution of overall DEGs among the different comparison groups i.e., 1T-2T, 1T-3T, and 2T-3T is represented using Circos plots (Figure 8).

### Differential Expression of Genes in Cultivars

**Figure 9:**
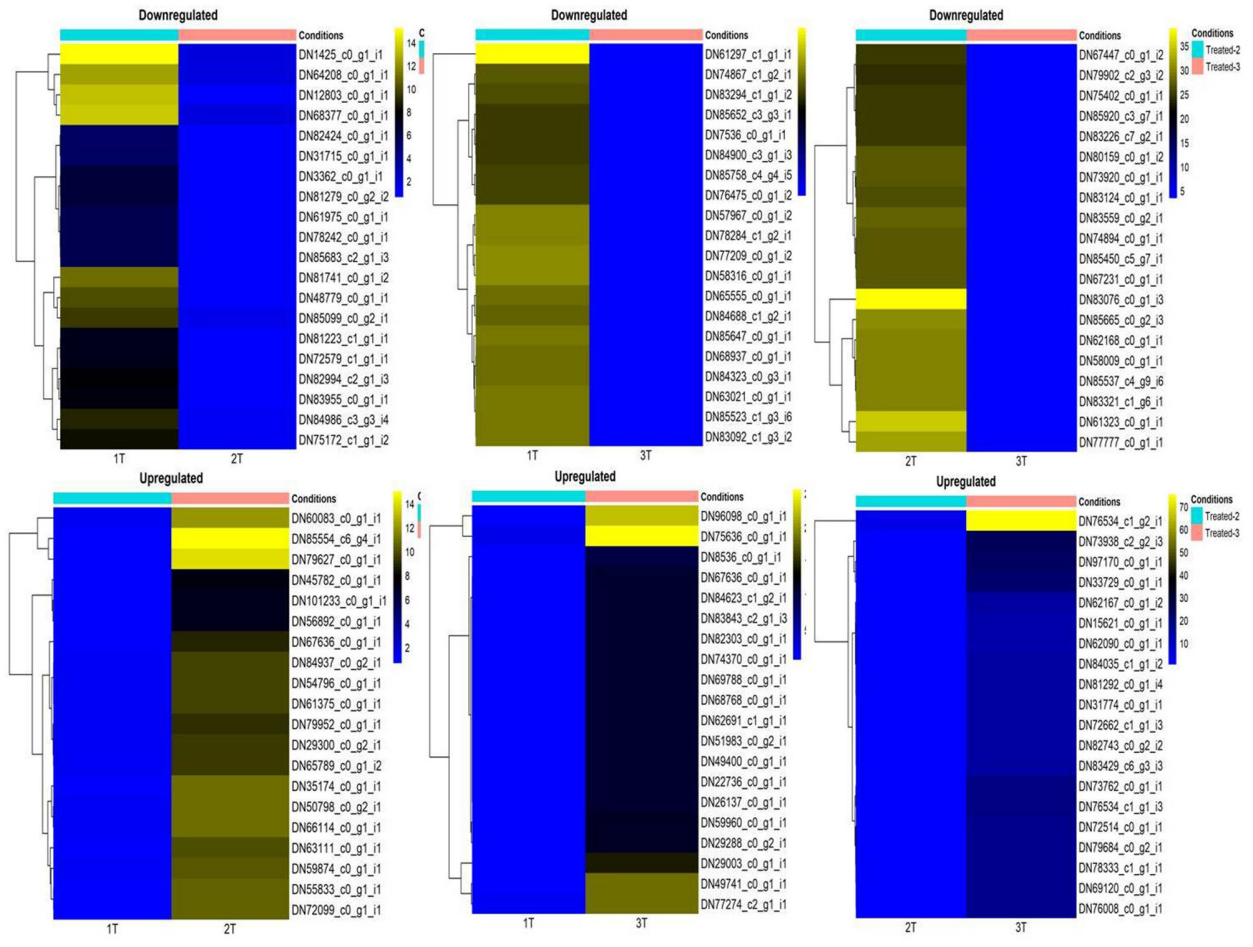
Significantly up- and down-regulated differentially expressed genes following Al-stress (148 μM AlCl_3_·H_2_O) in lentil genotypes as represented through heat map in combinations 1T-2T, 1T-3T, and 2T-3T, where, 1T: L-4602 treated, 2T: BM-4 treated, and 3T: ILWL-15 treated.

Differential expression of genes in different combinations under different conditions was deduced using the EdgeR package. Heat maps showing significantly up- and down-regulated DEGs for the different comparison groups are represented in Figure 9. When the tolerant cultivar was compared to the sensitive cultivar under Al-stress condition, significantly up-regulated DEGs with logFC > 5 were BTB/POZ domain-containing protein NPY1, PX domain-containing protein EREX, light-dependent short hypocotyls 1 protein, and ATP-dependent zinc metalloprotease FTSH 7 chloroplastic; whereas down-regulated DEGs included aldehyde dehydrogenase family 3 member F1, zinc-finger homeodomain protein 4, probable carboxylesterase 11, peptidyl-prolyl cis-trans isomerase 1, and G-type lectin S-receptor-like serine/threonine-protein kinase SD1-13 (Supplementary Tables 3, 4). Genes characterised as protein light-dependent short hypocotyls 1, PX domain-containing protein EREX, BTB/POZ domain-containing protein NPY1, and ATP-dependent zinc metalloprotease FTSH 7 chloroplastic were down-regulated with an absolute log_2_fold change > −5.

When compared with their control, the top significantly up-regulated DEGs in the Al-tolerant cultivar with Log_2_FC > 8 under Al-stress conditions were defensin-like protein 39, retrovirus-related pol polyprotein from transposon TNT 1-94, transcriptional activator DEMETER, threonine synthase chloroplastic, and ATP-binding cassette (ABC) transporter A family member 2; whereas significantly down-regulated DEGs with absolute log_2_FC > 7 were characterised as histone-lysine N-methyl transferase ATX4, protein RAE1, retrovirus-related pol polyprotein from transposon TNT 1-94, disease resistance protein RPP5, and probable serine/threonine-protein kinase PBL23. Likewise, the top up-regulated DEGs under Al-stress conditions with log2FC > 8 when the Al-sensitive genotype was compared with its respective control were auxin-responsive protein SAUR72, probable polygalacturonase, putative transposon Ty5-1 protein YCL074W, protein sodium potassium root defective 2, and protein CLEC16A homologue, while significantly down-regulated DEGs with absolute log_2_FC > 7 were amidophosphoribosyl transferase 2 (ATase 2) chloroplastic, AMP deaminase, protein chromatin remodelling 25, calcium uniporter protein 2 mitochondrial, and UDP-galactose/UDP-glucose transporter 3.

### Differential Expression of Genes Between Cultivars and Wild

**Figure 10:**
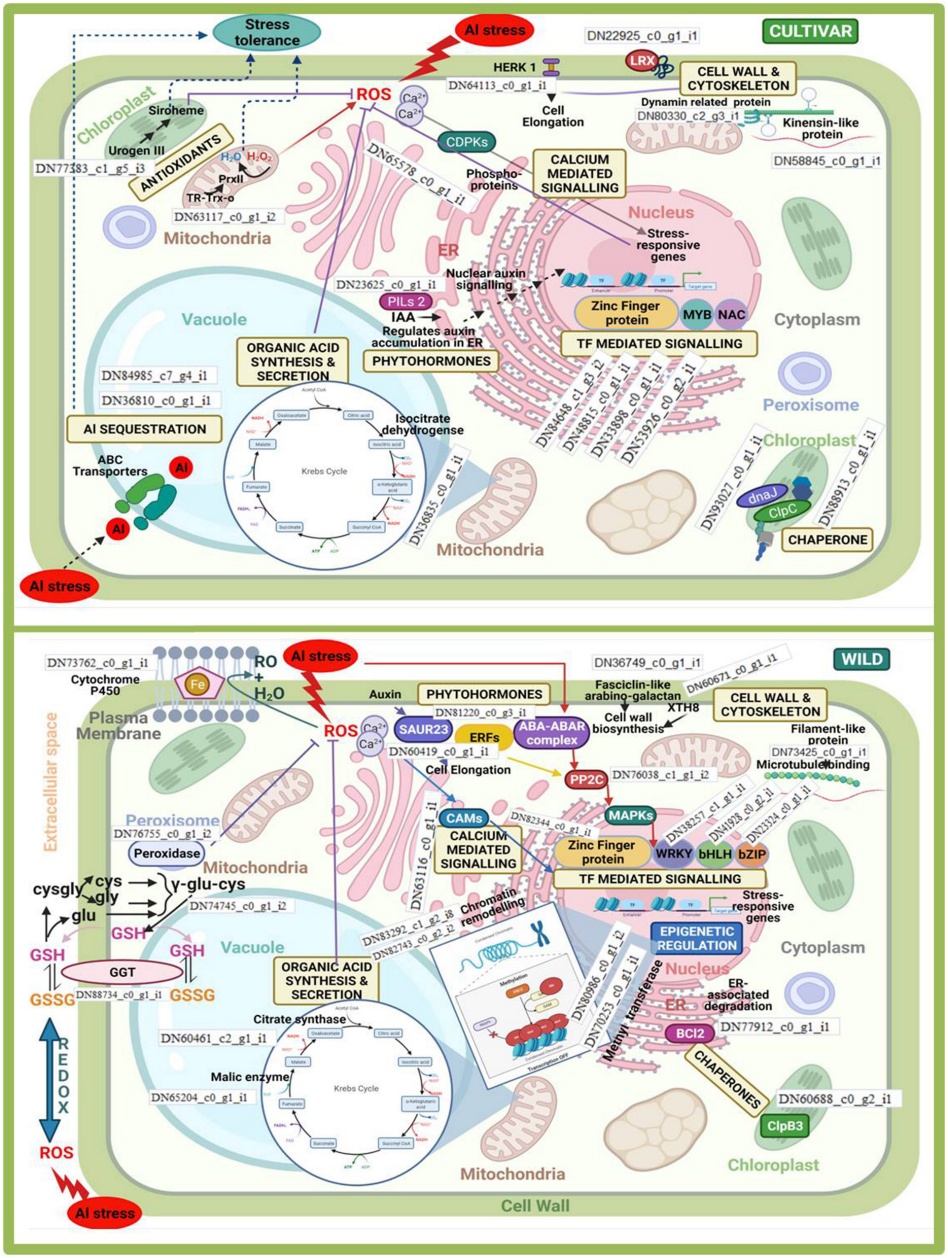
Inter- (wild vs. cultivars) and intra- (within cultivars) species comparison of differentially expressed genes associated with major aluminium tolerance pathways in lentil.

A comparison between the cultivated (L-4602 and BM-4) and wild (ILWL-15) genotypes for DEGs under Al-stress conditions revealed that most DEGs with absolute Log_2_FC change > 6 were found down-regulated (Figure 10). Furthermore, when Al-tolerant cultivar L-4602 and wild genotype ILWL-15 were compared, the top significantly up-regulated DEGs with Log_2_FC > 5 in the wild genotype were retrovirus-related Pol polyprotein from transposon TNT 1-94, DDB1- and CUL4-associated factor 8, polypyrimidine tract-binding protein homologue 2, Ycf49-like protein, and shaggy-related protein kinase theta. whereas the top down-regulated DEGs with absolute Log_2_FC > 7 included protein SRC2, uncharacterised protein ycf68, probable LRR receptor-like serine/threonine-protein kinase At3g47570, uncharacterised mitochondrial protein AtMg00310, and serine carboxypeptidase-like 40. Similarly, when the Al-sensitive genotype (BM-4) and the wild genotype (ILWL-15) were compared, the top significantly up-regulated DEGs in wild with Log_2_FC > 6 were protein SCO1 homologue 1 mitochondrial, ubiquitin carboxyl-terminal hydrolase 2, phosphatidylinositol 4-phosphate 5-kinase 1, fasciclin-like arabinogalactan protein 12, and subtilisin-like protease SBT 2.6. Similarly, the top down-regulated DEGs showing Log_2_FC > 7 in the wild genotype were exocyst complex component EXO70B1, vacuolar protein sorting-associated protein 54 chloroplastic, protein weak chloroplast movement under blue light 1, octanoyltransferase, and protein TPX2. Under Al-stress conditions, DEGs in the wild genotype, when compared with its control, were found to have a differential expression as high as Log_2_FC > 11. In the wild genotype, all the DEGs with absolute Log_2_FC > 7.2 were found to be up-regulated. Furthermore, the top up-regulated DEGs characterised in this comparison group with Log_2_FC > 9 included pentatricopeptide repeat-containing protein At3g23020, U-box domain-containing protein 13, 60S acidic ribosomal protein P1-2, auxin response factor 6, and GDSL esterase/lipase At1g29670. The top down-regulated DEGs with absolute Log_2_FC > −9 in the wild genotype were TMV resistance protein N, F-box protein At2g39490, putative oxidoreductase C1F5.03c, uncharacterised mitochondrial protein AtMg00810, and cyclin-dependent kinase F−1.

### GO Annotation of Contigs to the Reference Databases

Using publicly available databases for legumes, namely, *Cicer arietinum, Lotus japonicus, Madia sativa, Cajanus cajan, Medicago truncatula*, and *Glycine max*, and, publicly available protein databases such as Reference Sequence (RefSeq), Protein Database Bank (PDB), SWISS-PROT, The *Arabidopsis* Information Resource (TAIR), UNIREF100 (Uniport Reference), and Gene Ontology (GO), selected contigs were sequence aligned and are presented in Supplementary Figure 1. The maximum number of contigs (79,142) was found to be aligned to that of *M. trancatula* amongst the legume databases and the highest number of aligned databases was demonstrated by TAIR among the publicly available protein databases. The total number of unigenes found to be aligned with other model crops, namely, *Arabidopsis thaliana, M. truncatula*, and *G. Max*, was 7,476, 1,263, and 20,419 hits, respectively. The highest number of hits was found to be matched with the Genbank database (20,419). Details of SSRs and SNPs generated in different samples and the total number of reads used has been summarised in an Supplementary Table 5.

### qRT-PCR Endorsement

**Figure 11:**
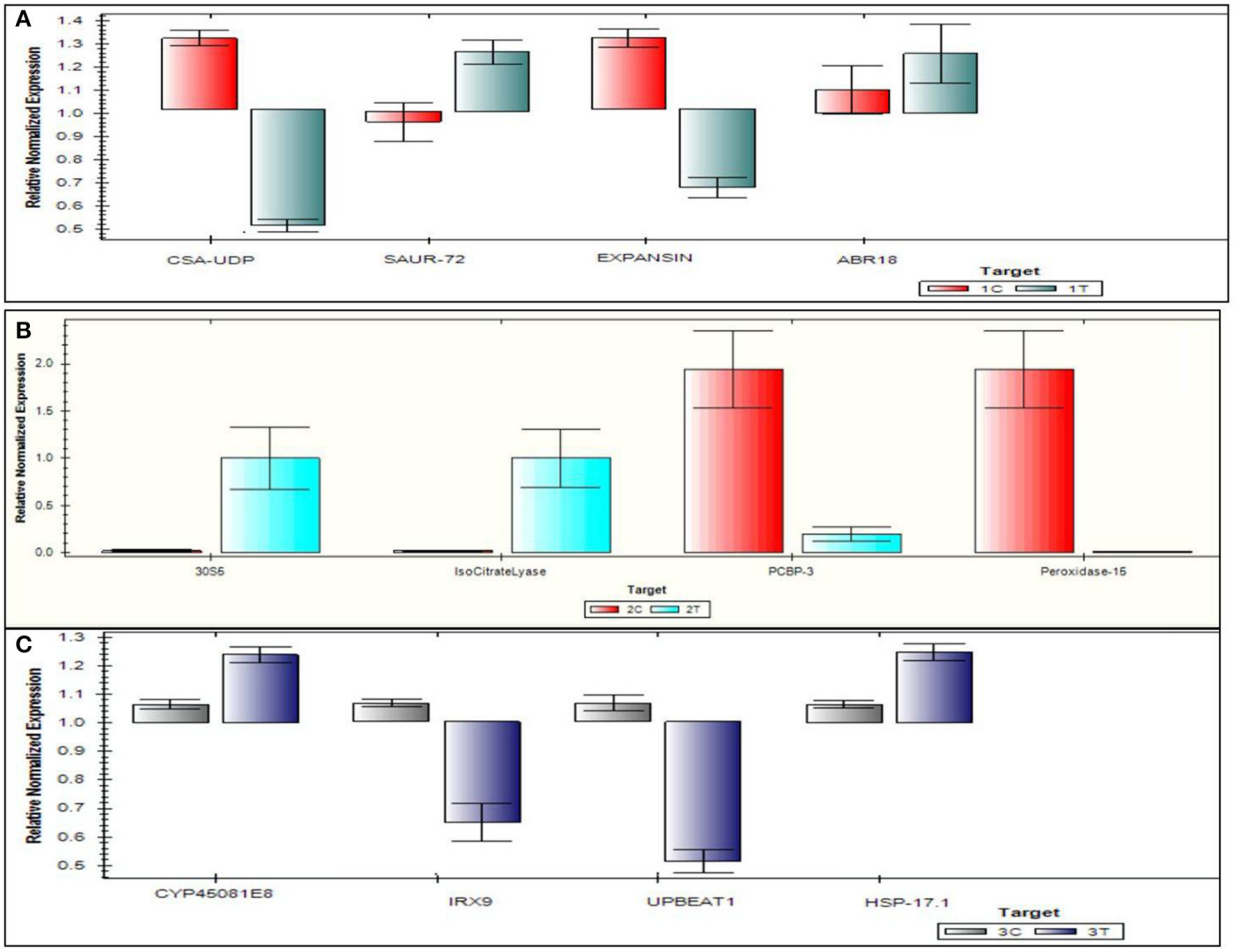
Quantitative real time polymerase chain reaction validation of 12 genes on six samples following Al-treatment (148 μM AlCl_3_·H_2_O) in lentil genotypes. **(A)** 1C-1T, **(B)** 2C-2T, **(C)** 3C-3T, where 1C: L-4602 control, 2C: BM-4 control, 3C: ILWL-15 control, 1T: L-4602 treated, 2T: BM-4 treated, and 3T: ILWL-15 treated.

The top four DEGs from each of the comparison groups, *viz*., 3C-3T, 2C-2T, and 1C-1T were used to validate DEGs. For the 1C-1T comparison group, SAUR-72, ABR 18, Expansin, and CSA-UDP were selected, where SAUR-72 and ABR 18 were found to be up-regulated and the other two genes were found to be down-regulated. For comparison group 2C-2T, isocitrate lyase, 30S5, peroxidase-15, and PCBP3 genes were selected, and all of them were found to be up-regulated; whereas for the 3C-3T group, HSP 17.1, IRX9, CYP45081E8, and UPBEAT1 were selected, of which CYP45081E8 and HSP 17.1 were found to be up-regulated and the other two genes were down-regulated. The qRT-PCR expression of selected top 12 genes is represented in Figure 11. A close similarity was observed when the log_2_ fold change data from the qRT-PCR analysis were compared with the RNA-seq data, which further confirms the differential expression of various genes under Al-stress conditions.

### Functional Characterisation of DEGs

For functional characterisation of DEGs, the transcripts were assigned with GO annotation terms. For combinations 1T-2T, 2T-3T, and 1T-3T, a total of 2,857, 2,872, and 3,403 GO annotation terms were found, respectively (Supplementary Figure 2). When the three genotypes were compared with their corresponding controls, significantly enriched GO annotation classes for DEGs were found in all the comparison groups within the cellular component category comprising membrane, cell junction, supramolecular complex, organelle, symplast, extracellular region, protein containing complex, and membrane enclosed lumen. In the molecular function category, most significant DEGs were involved in transporter activity, binding, molecular transducer, catalytic activity, antioxidants, molecular function regulator, structural molecule activity, and transcription regulation. In this category, signal transducer activity was absent in the 1C-1T group. Moreover, in the wild genotype, it was found only under treatment conditions and not under control conditions; whereas in the biological process category, maximum DEGs were involved in metabolic processes, biogenesis, biological regulation, localisation, cellular processes, response to stimulus, multi-organismal processes, reproductive processes, developmental processes, signalling, growth, immune system processes, and rhythmic processes. In this category, the cell proliferation process was found to be enriched only in the 2C-2T and 3C-3T comparison groups.

**Figure 12:**
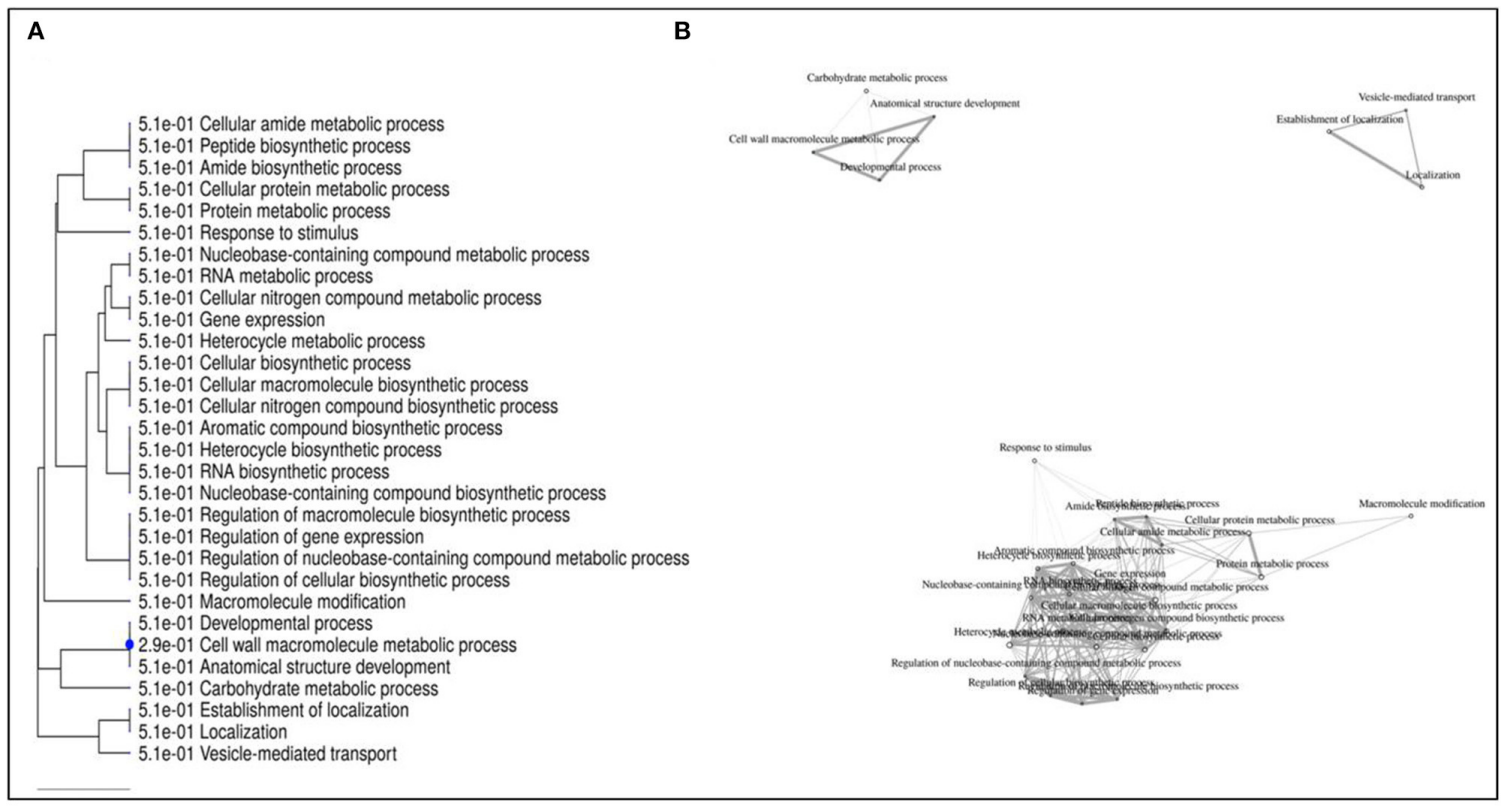
Gene ontology (GO) annotation tree constructed using 50 differentially expressed genes following Al-stress in lentil genotypes.

Further enrichment analysis of the top 50 DEGs selected from all the comparison groups were also performed and presented through a GO annotation tree (Figure 12), which revealed three major enrichment categories. Category I included four interconnected processes, *viz*., carbohydrate metabolic process (1,233 genes), cell wall macromolecule metabolic process (144 genes), anatomical structure development (1,639 genes), and developmental processes (1,666 genes). Category II was found to be involved with vesicle mediated transport (283 genes), establishment of localisation (2,343 genes), and localisation (2,373 genes); whereas category III was found to be involved in interconnected functional processes (66,289 genes) of which several metabolic processes are associated with cellular nitrogenous compounds, nucleobase-containing compounds, proteins, RNA, cellular amides, along with several biosynthesis processes associated with cellular processes, along with gene expression regulation as well as metabolic processes. Furthermore, gene expressions related to stimulus response generation and macromolecular modifications were also found.

Further functional analysis *via* a proteomics study showed that cell wall biosynthesis- and modification-related differentially abundant proteins (DAPs) were higher in the Al tolerant genotypes when compared with the Al-sensitive one. The details of DEGs with their corresponding DAPs are listed in Table 2.

Table 2Differentially expressed genes (DEGs) and differentially abundant proteins (DAPs) related to cell wall biosynthesis and modifications in combination 1T-2T.

**Table:** 

**ID**	**logFC**	**logCPM**	**PValue**	**FDR**	**DescriptionSP**
**DEG related to cell wall biosynthesis and modifications in combination 1T-2T**					
TRINITY_DN80330_c2_g3_i1	2.90	−0.1371	1.52E−05	0.001686	Dynamin-related protein 1E
TRINITY_DN75722_c0_g1_i1	2.43	0.249257	2.83E−06	0.000593	Glucan endo-1 3-beta-glucosidase 13
TRINITY_DN58845_c0_g1_i1	2.43	0.247298	2.83E−06	0.000593	Kinesin-like protein KIN-4C
TRINITY_DN22925_c0_g1_i1	2.41	−0.44115	0.00149	0.02834	Leucine-rich repeat extensin-like protein 4
TRINITY_DN67230_c0_g1_i1	1.07	0.700075	0.007149	0.073112	Expansin-A1^3^

**Table:** 

**Master**	**Accession**	**Description**	**Found in Sample: [S4] F4: Sample**	**Found in Sample: [S2] F2: Sample**
**DAP related to cell wall biosynthesis and modifications in combination 1T-2T**				
Master Protein	A0A072VL62	125 kDa kinesin-like protein OS = Medicago truncatula OX = 3880 GN = 25484095 PE = 3 SV = 1	Peak Found	Medium
Master Protein	A0A0R0KHR2	(1–>3)-beta-glucan endohydrolase OS = Glycine max OX = 3847 GN = GLYMA_04G218600 PE = 3 SV = 1	Medium	Medium
Master Protein	A0A072VCC8	Expansin-like protein B1 OS = Medicago truncatula OX = 3880 GN = 25487639 PE = 3 SV = 1	Peak Found	Medium
Master Protein	G7LAV9	Extensin, putative OS = Medicago truncatula OX = 3880 GN = 11440896 PE = 4 SV = 1	Not Found	Medium
Master Protein	I1KNS5	Dynamin GTPase OS = Glycine max OX = 3847 GN = 100809385 PE = 3 SV = 2	Not Found	Medium

*DAPs: differentially abundant proteins, DEGs: differentially expressed genes, 1T: L-4602 treated, 2T: BM-4 treated*.

### Pathway Analyses Under Al-Stress Conditions

#### Comprehensive Pathway Analysis

**Figure 13:**
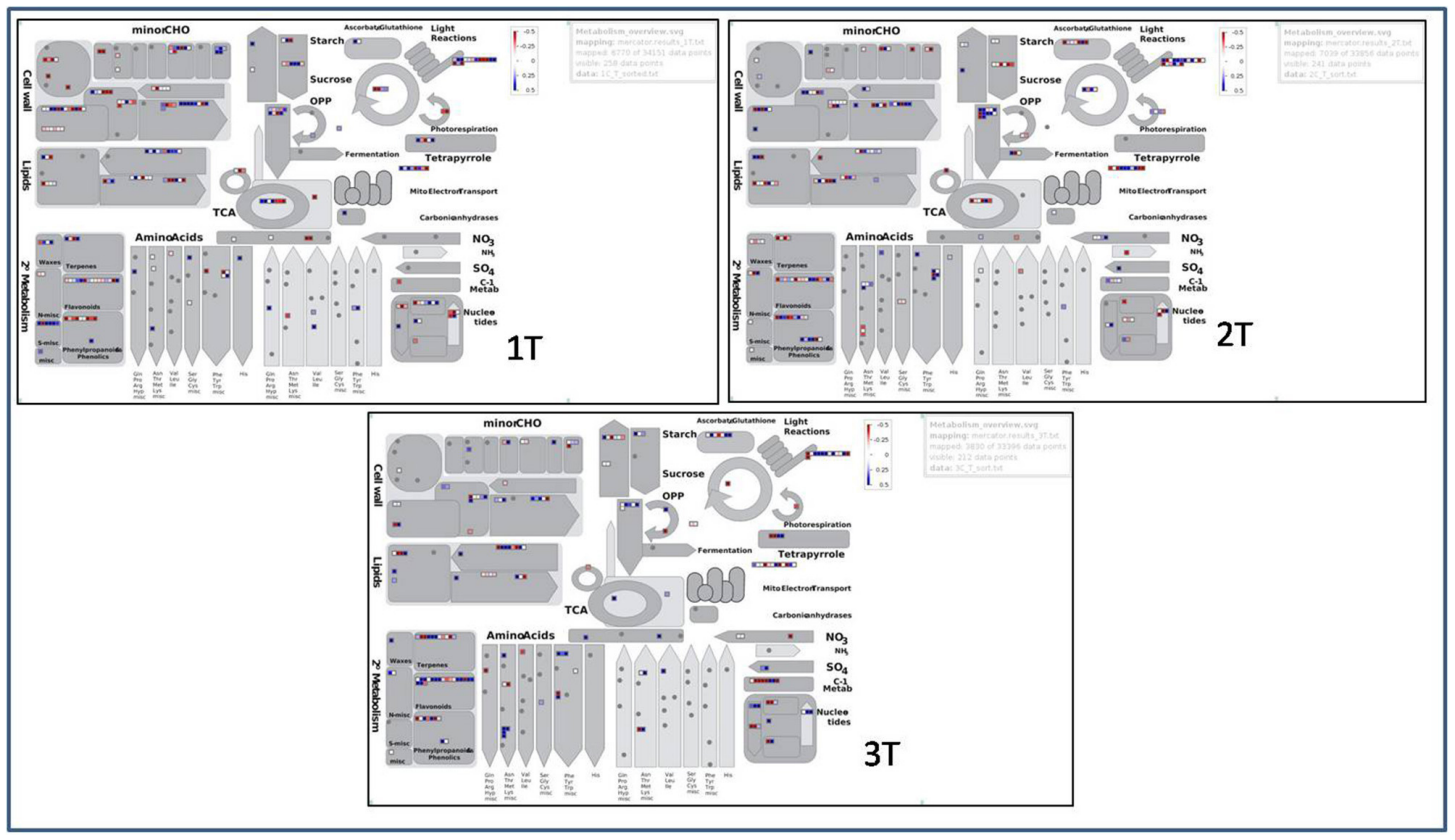
MapMan representation of up- and down-regulated BINs in tolerant (L-4602, ILWL-15) and sensitive (BM-4) lentil genotypes under aluminium stress conditions.

The analysis of pathways which are involved in regulation of aluminium stress in cultivars and wild was represented using up- and down-regulated BINs visually illustrating genes pertaining to functional pathway using MapMan software (Figure 13). There were a total of 6,670, 7,039, and 3,830 data points generated from 34,151, 33,856, and 33,396 DEGs for samples 1T, 2T, and 3T under Al-stress conditions, respectively. Altogether, all the studied samples showed a similar style of visual representations (BINs). However, BINs representing the cell wall were more prominent in the tolerant genotypes compared with the sensitive and wild genotypes under Al-stress conditions. The number of BINs that were characterised as nucleotide pathways, as well as photorespiration, differed within the tolerant and sensitive genotypes. Furthermore, the BINs pertaining to secondary metabolites, e.g., flavonoids, terpenes, etc., were higher in the wild sample when compared with the tolerant and sensitive cultivars. The pathways for Al-tolerance in lentil genotypes was devised using data generated from the investigation of top 135 DEGs in all the six comparison groups. The differential gene expression analysis showed 63 novel and uncharacterised proteins involved under Al stress conditions. The most prominent pathway highlighted in this analysis involved genes belonging to organic acid anion synthesis and exudation, phytohormone signalling genes, Al-induced ROS detoxification through enzymatic activity, Al-induced callose synthesis, and an alternate pathway involving metacaspases.

#### Intra- and Interspecies Pathway Analysis

Differentially expressed genes that significantly provided tolerance to Al stress in both cultigens and wild were associated with pathways such as cell wall and cytoskeleton synthesis, calcium-mediated signalling, organic acid synthesis and secretion, production of phytohormones, transcription factor (TF)-mediated signalling, and protein modifications involving different chaperones. However, most of these pathways were influenced by different genes when intra- and interspecies comparisons were made. The involvement of corresponding DEGs within these pathways is schematically represented in Figure 10. Moreover, some of the pathways were specifically influenced either in the cultivars or the wild under Al^3+^ stress conditions. Al^3+^ ions sequestering within the vacuole was specifically observed in the case of the cultivars, whereas epigenetic regulation categorically enhanced Al^3+^ tolerance in wild. In the case of the former, the upregulation of DEGs related to abscisic acid (ABA) transporters helped in sequestering Al^3+^ ions in vacuoles; whereas those involved in chromatin remodelling and transfer of methyl groups participated in the latter.

Under Al^3+^ stress condition, cultivars showed differential expression of Uroporphyrinogen-III synthase chloroplastic and Thioredoxinreductase2. These genes helped in in reducing the effect of Al-induced ROS. In case of wild, this function was taken care of by upregulation of GGT and peroxidase. Similarly, in the cultivars, organic acid synthesis was upregulated majorly by the involvement of isocitrate dehydrogenase, whereas malate and citrate syntase enzymes were majorly upregulated in the wild genotype. Organic acid anion transporter MATE was found to be significantly up-regulated in the tolerant genotype when compared with the sensitive one, whereas when the moderately tolerant wild genotype was compared with the cultivated tolerant genotypes, putative multidrug resistance protein, ALMTs 7 and 12, and organic acid synthesis genes such as aconitate hydratase (Aco), acetyl-CoA carboxylase, acetyl-CoA acetyl transferase, succinate dehydrogenase, and enoyl-CoA hydratase, were found to be up-regulated. Cell wall and cytoskeleton synthesis showed the major involvement of dynamin-related proteins and kinensin-like proteins in the cultivars. Similarly, fasciclin-like arabino-galactan was utilised in the case of the wild genotype. DEGs associated with CDPKs and CAMs, which were involved in calcium-mediated signalling, were majorly upregulated under Al^3+^ stress conditions in the cultivars and the wild genotype, respectively. Chaperones mediated folding of protein in wild also involved a totally distinct gene clp83, while in case of cultivars a couple of genes viz. dnaJ and clpC were also involved. Al^3+^ stress also influenced the phytohormone pathway, where auxin-induced SAUR23 and ERFs, together with the ABA-ABAR complex, activated PP2C, which mediated MAPK signal transduction to activate TF WRKY (in particular) in case of the wild genotype, whereas in case of cultivars, PILs were involved in the regulation of auxin accumulation in the endoplasmic reticulum (ER). Thus, in the cultivars, the increase in auxin acted as a nuclear auxin signal that induced TF-mediated signalling to regulate stress-responsive genes. TFs such MYB and NAC were majorly induced in the cultivars, whereas WRKY and bHLH mainly promoted Al^3+^ stress tolerance in the wild genotype. Zinc finger protein TF was induced in both the cultivars and wild genotype. Apart from that, when tolerant genotypes were compared with sensitive one, callose synthesis was increased in former due to the up-regulation of callose synthases 3, 7, and 12 in the roots. In addition to the mechanism for detoxification of ROS and callose synthesis, an alternate pathway was found to be operated in the tolerant genotypes (wild or cultivated) involving metacaspases 1, 4, and 9 to induce programmed cell death (PCD) under Al-stress conditions.

## Discussion

The evaluation of Al^3+^ tolerance in crop plants is imperative for breeding crops to improve Al resistance. Root growth inhibition is a well-documented trait for the evaluation of Al tolerance in crop plants exposed to Al^3+^ stress for short and long durations (Singh et al., 2012, 2015, 2016). The previous studies (Singh et al., 2012, 2015, 2016) suggested that 148 μM Al^3+^ is the optimal Al^3+^ ion concentration in evaluating lentil genotypes for Al tolerance. Because of significant differences in root growth in contrasting genotypes at this concentration, same concentration was utilised in this study as well. The impairment of physio-biochemical attributes in response to long-term exposure to Al^3+^ is also demonstrated in the previous studies. Therefore, in this analysis, to compare the genotypes based on RRE, short-term exposure with 148 μM Al^3+^ was given to the genotypes for 3, 6, 12, and 24h. The seedlings of Al-tolerant and wild genotypes showed > 50% RRE as compared with the sensitive genotype within 6 h of exposure to Al stress. It has also been observed that a clear differentiation between the Al-tolerant and Al-sensitive genotypes could be detected based on the accumulation of Al, callose, and proline, together with the induction of antioxidant activities, at 6 h of exposure to Al^3+^ stress (Figures 2, 4). Therefore, a minimum of 6 h Al^3+^ exposure is important for the commencement of symptoms for Al^3+^ toxicity, which has also been suggested in various studies (Ahn et al., 2002; Ma et al., 2004). Previous findings have reported impaired root growth at 6 h of Al^3+^ exposure in *Cucurbita pepo* (Ahn et al., 2002) and increased citrate secretion in barley (Ma et al., 2004).

The impairment in physio-biochemical traits was utilised to differentiate Al-tolerant from sensitive genotypes in this study. Increased accumulation of callose and Al^3+^ in roots of the sensitive genotype was observed, which is in line with the results of previous studies on mung bean (Singh et al., 2015) and lentils (Singh et al., 2016). The plausible explanation for the low accumulation of Al^3+^ and callose in the tolerant genotype must be the high exclusion of Al^3+^ through root tips by secretion of organic acids or their anions, as reported in the previous study (Singh et al., 2016). The Al-tolerant genotype also exhibited higher activities of ROS-detoxifying enzymes, such as SOD, APX, and GPX, and thus evaded the oxidative damage caused by toxic Al^3+^ ions. Similar findings were also reported in the previous study (Singh et al., 2016). The increase in total sugar and starch contents of the lentil genotypes under Al stress conditions are in line with the results reported by Samad et al. (2020), Moreno-Alvarado et al. (2017), and Mishra and Dubey (2008) in the case of rice genotypes. They suggested that Al toxicity impairs starch and sugar metabolism and favours the accumulation of hexoses by enhancing activities of sucrose-hydrolysing enzymes. On the contrary, in the case of *Quercus serrata* Thunb., reduction in starch and soluble sugars under Al-stress conditions was reported (Moriyama et al., 2016).

Aluminium stress inhibits water uptake by roots as it interacts with membrane components and modifies its structural properties such as fluidity and permeability as well as ionic environment near the surface of the cell (Yang et al., 2013; Ali, 2017). This interaction causes a reduction in MSI, as evidenced in this study, as well as in the study on mungbean plants by Ali (2017).

The root tips of the genotypes were further assessed for any cytological changes, such as chromosomal aberrations under Al-stress conditions, and revealed chromosomal fragmentation, stickiness, bridges, and coagulation at metaphase and anaphase, together with nuclear lesions. Chromosomal aberrations due to disruptions in mitotic cycles of cells can explain the reduced root growth under Al stress conditions. Similar results were also reported by Li et al. (2015) and Zhang et al. (2014), who also observed chromosome bridges at anaphase and sticky chromosomes in Al^3+^-treated root tip cells of sunflower and *Pinus*, respectively. Similarly, Jaskowiak et al. (2018) observed that Al stress significantly reduced the mitotic activity in root tip cells of barley and induced micronuclei in the cells. The physio-biochemical and cyto-morphic analyses revealed the harsh effects of Al^3+^ ions under short-term exposure conditions in all the lentil genotypes; however, the sensitive genotype was the most affected by Al-induced toxicity.

Variation in Al tolerance caused by the differential expression of genes, which segregates in contrasting varieties, is termed as the expression level polymorphism (ELP) (Delker and Quint, 2011). Characterisation of ELP *via* transcriptomics is a favourable approach to spot novel Al-tolerant genes. Therefore, a *de novo* transcriptomic analysis was performed in this study to delineate the differential expression of genes among the cultivars, together with the analysis between the cultivars and wild genotypes in response to Al stress. Similarly, in a study by Kusunoki et al. (2017), the ELP in *Arabidopsis* accessions helped in the identification of a series of Al-tolerance candidate genes during root growth.

In this study, the top significantly up-regulated DEGs were found to be involved in defence, cellular growth, development, protein degradation and transport, etc. Gene encoding for protein EREX was highly up-regulated and was involved in controlling membrane trafficking and vacuolar transport of storage proteins (Sakurai et al., 2016). Light-dependent short hypocotyls 1 protein was another major DEG said to be involved in developmental regulation and stimulation of cellular growth in response to continuous, far-red and blue lights (Zhao et al., 2004). Protein NPY1 containing BTB/POZ domain is highly up-regulated under Al stress conditions, and it acts as an adapter of an E3 ubiquitin-protein ligase complex, which is involved in the degradation and ubiquitination of the targeted proteins. Another significantly up-regulated DEG under Al stress conditions is ATP-dependent zinc metalloprotease FTSH7, which is believed to be involved in the exclusion of damaged D1 protein in photosystem II and thylakoid formation, thus, preventing cell death associated with high-intensity light (Sakamoto et al., 2003; Zelisko et al., 2005). Similarly, significantly down-regulated DEGs with suppressed functions in the tolerant cultivars compared with the sensitive one were found in this analysis. DEGs that showed down-regulation included zinc-finger homeodomain protein 4, a presumed transcription factor believed to be involved in the regulation of floral induction (Torti et al., 2012). Aldehyde dehydrogenase and carboxylesterase family members have catalytic roles, and protein kinase SD1-13 is involved in the repression of disease resistance signalling pathways (Kim et al., 2009).

When compared with its control, the top significantly up-regulated DEGs in the tolerant cultivars were found to have catalytic functions (TNT 1-94) and anti-fungal activity (Defensin-like protein 39). DEMETER is reported to be involved in gene imprinting and acts as transcriptional activator for floral and vegetative development (Choi et al., 2002, 2004; Morales-Ruiz et al., 2006). Threonine synthase chloroplastic is involved in the formation of L-threonine, whereas ABC transporter A family member 2 showed nucleotide binding. The down-regulated DEGs included genes involved in catalysis, *viz*., TNT 1-94, ATX4, and RAE1 protein involved in ubiquitin binding, RPP5 protein involved in disease resistance, and plant defence signalling protein kinase PBL23.

When wild (ILWL-15) and tolerant cultivar (L-4602) were compared, those DEGs that were significantly up-regulated in wild genotype were found to be attributed in regulation of transcription process in growing cells; protein ubiquitination, pre-mRNA splicing, and catalysis, etc. Likewise, when the sensitive cultivars were compared to its control, the top up-regulated DEGs were involved in cell expansion, auxin transport, root meristem patterning, etc. Genes encoding for sodium potassium root defective 2 protein were significantly up-regulated. Sodium potassium root defective 2 protein is found to be involved in the binding of metals, thus, affecting the root growth of the sensitive genotype under Al stress conditions. The down-regulated DEGs also included genes that were involved in DNA repair and mitotic chromosomal recombination (chromatin remodelling 25) (Shaked et al., 2006), cell division, and chloroplast biogenesis (amido phosphoribosyl transferase 2 chloroplastic) (Hung et al., 2004).

When the Al-sensitive cultivar (BM-4) was compared with the tolerant wild genotype (ILWL-15), significantly up-regulated DEGs were involved in the catalysis (Westergren et al., 2001), cellular redox homeostasis (Attallah et al., 2011), and processing of ubiquitinated proteins (Yan et al., 2000), etc; whereas down-regulated DEGs in the wild genotype included protein TPX2, which exhibits a microtubule binding activity and regulates the onset of mitosis (Vos et al., 2008; Petrovská et al., 2013). This explains the reduced mitotic activity in root tip cells under Al-stress conditions.

Similarly, differential gene expression analysis of the wild genotype with its control was performed and top up-regulated DEGs included 60S acidic ribosomal protein P1-2 and U-box domain-containing protein 13, which are involved in protein synthesis. Auxin response factor 6 was another major DEG that is involved in reproductive function regulation involving jasmonic acid stimulation, and stamen and gynoecium maturation, etc. (Hagen and Guilfoyle, 2002; Nagpal et al., 2005). Amongst the top down-regulated DEGs, TMV resistance protein is characterised to be involved in disease-resistance signalling, while cyclin-dependent kinase F1 modulates activities of other kinase proteins by phosphorylation (Tsakraklides and Solomon, 2002). Interestingly, all the top DEGs in all the comparison groups were involved in the down-regulation of several disease-resistant genes, which further confirms the cytotoxicity conferred by Al^3+^ ions in lentils.

To validate the data generated from the different comparison groups, the top 12 DEGs from the different comparison groups were utilised. Most of the DEGs that were utilised for the validation study were involved in developmental functions, root meristem patterning, and auxin transport (SAUR-72), etc., in the 1C-1T group. Genes, such as 30S5, isocitrate lyase, poly(C)-binding protein 3 (PCBP3), and peroxidase-15, were found to be up-regulated in the 2C-2T group and are known to be involved in dessication tolerance (isocitrate lyase) (Jeon et al., 2015), post-transcriptional regulations (PCBP3) (Choi et al., 2009), and checking levels of H_2_O_2_ that non-enzymatic disintegrates the cell wall (peroxidase-15) (Souza et al., 2002; Cordoba-Pedregosa et al., 2005). In the 3C-3T comparison group, genes CYP45081E8 and HSP 17.1 were found to be up-regulated, while IRX9 and UPBEAT1 were down-regulated. CYP45081E8 is a monooxygenase (probable) involved in the oxidation of isoflavones (Liu et al., 2003), whereas HSP17.1 belongs to a small heat shock protein (HSP20) family, which is known to be involved in abiotic stress signalling (Lopes-Caitar et al., 2013). The down-regulated IRX9 gene is involved in secondary cell wall synthesis (Faria-Blanc et al., 2018), and UPBEAT1 is a TF involved in root growth differentiation by curbing peroxidise expression in root elongation zones (Tsukagoshi et al., 2010). Most DEGs in this analysis were found to be involved in cellular growth, root growth, protein transport and auxin signalling. After validating the DEGs, putative pathway analysis for Al tolerance was performed to get an overview of Al tolerance mechanisms in lentils.

The overall pathway analysis under Al-stress conditions reveals that in the tolerant genotypes the most prominent BINs were related to the cell wall. The enhanced up-regulation of genes related to cell wall synthesis in the Al-tolerant genotype (L-4602), when compared with the sensitive ones, confirms their involvement in Al^3+^ resistance. The reduced growth and other cell wall-associated genes in the sensitive genotype can be explained by the high affinity of Al^3+^ ions for cell wall, which causes increased cell wall rigidity (Horst et al., 2010) and, therefore, leads to reduced cell wall growth and cell elongation (Blarney et al., 2004; Xu et al., 2018). Schmohl and Horst (2000) reported that the ability of Al^3+^ to bind pectin present in the cell wall determines its capability to inhibit root growth. This finding further confirms the observations made in this study. Several pectin modification and synthesis-related genes, such as pectinesterase inhibitor 41 and probable arabinosyl transferase ARAD1, were found in this study. Also, the release of secondary metabolites, such as flavonoids and organic acids, from the root apex is another well-established regulatory mechanism for Al resistance (Delhaize et al., 2012). Increase in BINs related to secondary metabolites in the wild genotype improved Al^3+^ tolerance because of the production of various secondary metabolites. Furthermore, the pathway analysis involving the top 135 DEGs from all the comparison groups revealed various uncharacterised and novel Al-stress response-related genes involved in organic acid anion synthesis and its exudation, generation of phytohormone response, ROS detoxification, biosynthesis of callose, and alternate pathway genes coding for metacaspases. Function correlation showed DEGs and DAPs, such as kinesin-like protein, (1->3)-beta-glucan endohydrolase, expansin-like protein, extensin, and dynamin GTPase related to cell wall biosynthesis and modifications, to be higher in the tolerant genotypes under Al stress conditions when compared with the sensitive one. A similar approach to detect potential regulator related to wheat root cell wall under Al stress conditions was reported by Yang et al. (2018).

Plants secrete organic acid or organic acid anions from root apices that chelate Al^3+^ ions and prevent them from binding to the cell membrane. PDK, which is crucial for intermediary metabolism and is involved in organic acid synthesis (Jan et al., 2006), is found to be up-regulated in this study. Organic acid synthesis genes were found to be up-regulated in the tolerant genotypes over the sensitive genotype. Similarly, in soybean, organic acid synthesis due to Al^3+^ stress in roots was more profound in a tolerant genotype over a sensitive genotype (Huang et al., 2017). Likewise, organic acid anion transporters for, e.g., Al-activated malate transporter (ALMT) as well as multidrug export protein AcrE (MATE) transporters, were found to be up-regulated significantly in the tolerant genotypes when compared with its respective control and play a crucial role in organic acid exudation and Al tolerance. Many transporters have been identified in different plants in response to ionic stresses, such as ALMT 9 that mediates vascular malate uptake (De Angeli et al., 2013); *Vv*ALMT 9 gene was found to be involved in the accumulation of organic acid anions such as malate and tartrate in vacuoles of grape berries (Conde et al., 2007); ALMT 12 activates anion channels mainly for chloride and nitrate ions transport (Meyer et al., 2010), etc. When the Al-tolerant and Al-sensitive genotypes were compared, the up-regulation of MATE gene was found to be significant in the tolerant genotypes. Similarly, in other studies on different crops, MATE family transporters were found to be involved in the exudation of organic acids under Al stress conditions. For example, Furukawa et al. (2007) reported a MATE family gene called *Hv*AACT1 in barley, which is known to be involved in the exudation of citrate under Al-stress conditions, and Xu et al. (2018) also noted the differential expression of MATE proteins under Al-stress conditions in maize.

Apart from organic acid synthesis and exudation, Al stress modifies the function of the plasma membrane through the activation of highly toxic ROS, which is mitigated by the activation of ROS-detoxifying enzymes. A comparison between moderately tolerant wild genotypes and the tolerant cultivars showed significant up-regulation of the ROS-mediated antioxidant signalling pathway involving APX and GPX as primary anti-oxidant enzymes involved in the detoxification of hydrogen peroxides. APX is a highly regulated enzyme involved in the detoxification of ROS (Saxena et al., 2011). Similar to this study, we identified a significantly high activity of GPX in wild lentil genotypes (Singh et al., 2016). Also, Fe-SOD genes were reported to be up-regulated in roots of aspen under Al^3+^ stress conditions (Yang et al., 2012).

Another major signalling pathway involved in Al-stress tolerance was the phytohormone signalling pathway, which activates many Al-resistance genes or suppresses root growth under Al-stress conditions (Daspute et al., 2017). Genes related to phytohormone signalling pathways were found to be up-regulated in the tolerant genotypes, providing resilience to Al^3+^ stress. Zhang et al. (2018) reported that reduced auxin synthesis is associated with Al-sensitivity in maize, which is in line with the results of this study. The accumulation of auxin causes root elongation, which is accompanied by enhanced synthesis of ethylene hormone, which, in turn, regulates auxin synthesis and its transport to root apices (Muday et al., 2012). Similarly, Yang et al. (2017) reported the up-regulation of Al stress-induced ethylene synthesis in *Arabidopsis*. In this investigation, ethylene-responsive TF (ERF113) is up-regulated in the wild genotype; it belongs to the AP2/ERF family and plays an important role in the ethylene signalling pathway (Dong et al., 2015).

Another major Al tolerance response is the deposition of callose in roots. Callose is a cell wall-linked polysaccharide that is considered to be a preliminary indicator of Al-stress-induced phytotoxicity and hampers root growth in plants (Zhang et al., 2015). Al-induced callose accumulation occurs adjacent to plasmodesmata, which destroy cellular communication and, thus, hamper root growth as well as water and nutrient uptake (Sivaguru et al., 2000). Al-induced callose deposition was much higher in the Al-sensitive genotype compared with the tolerant genotypes the wild genotype, which further explains the poor growth of the sensitive genotype under Al-stress conditions.

The alternate pathway involved in programmed cell death through the action of type I metacaspase (metacaspase 1) and type II metacaspases (metacaspases 4 and 9) was also found in the tolerant genotypes (whether tolerant cultivar or wild) in this study. Metacaspase 1 positively regulates PCD and cleaves specific substrates with conserved caspase-like putative catalytic residues, whereas metacaspase 4 regulates biotic and abiotic stress-induced PCD, and it is not involved in cleaving caspase-specific substrates (Watanabe and Lam, 2005). As per a microarray study performed on poplar, enzyme metacaspase 9 was found to play a major role in xylem PCD (Courtois-Moreau et al., 2009). Calcium-mediated signalling also played a role in influencing stress-responsive genes to improve Al^3+^ stress resilience *via* the upregulation of CAMs and CDPKs. Previously, Al^3+^ was reported to affect cellular calcium homeostasis in root hairs of *Arabidopsis* (Jones et al., 1998).

The differential expression of Al-tolerant genes in lentils confirms the Al tolerance mechanism as a highly regulated network of genes. The inter- and intraspecies comparison revealed that most of the above-discussed pathways were influenced by Al^3+^ stress in both cultivars and wilds. These pathways were controlled by TF-mediated signalling, where zinc finger TF affected Al^3+^ stress resilience in the cultivars as well as wild. ART1 and ATF1 zinc finger TFs were previously reported to regulate Al tolerance in rice and barley, respectively (Yamaji et al., 2009; Tsutsui et al., 2011; Wu et al., 2020). Later, in the case of rice, functional characterisation of ART2, which is the homologue of ART1, revealed that these two zinc finger TF homologues regulate different Al^3+^ tolerance pathways, where the former plays a supplementary role in Al^3+^ tolerance (Che et al., 2018). Zinc finger TF STOP1 was significantly up-regulated in the Al-tolerant lentil genotype than the Al-tolerant wild or sensitive genotypes. STOP1 plays a significant role in the regulation of downstream genes and is conserved in land plants (Iuchi et al., 2007). In *Arabidopsis*, the *At*STOP1 protein was found to be involved in the positive regulation of Al-tolerance genes for e.g., *At*ALMT1, *At*ALS3, and *At*MATE (Sawaki et al., 2009). It is also reported that SUMOylation of STOP1 regulates Al^3+^ tolerance in *Arabidopsis* (Fang et al., 2020). MYB and NAC TFs were majorly involved in the tolerance mechanism in the case of cultivars, whereas in the case of wild, WRKY and bHLH TFs were majorly involved. Zhou et al. (2016) found that MYB and WRKY TFs were involved in the regulation of ROS and flavonoid biosynthesis under Al^3+^ stress conditions in alfalfa. Similarly, zinc finger, MYB, and WRKY TFs, together, played critical roles in Al^3+^ stress resilience in tea (Huang et al., 2021). Li et al. (2020) reported that WRKY confers Al^3+^ tolerance by regulation of cell wall-modifying genes. Also, it was observed that the silencing of *OsWRKY22* escalated sensitivity towards Al^3+^ stress, since the TF was responsible for enhanced citrate secretion in rice (Li et al., 2018). Escobar-Sepúlveda et al. (2017) found that phytohormones play a key role in the regulation of NAC TFs under Al^3+^ stress conditions, which favours the growth and development of plants under Al^3+^ toxic conditions. Similarly, in the case of flax, under Al^3+^ stress conditions, this class of TFs was involved in regulating plant growth and development, together with the regulation of enzymes participating in cell wall modifications (Krasnov et al., 2019). Genome-wide analysis in tomato has identified 93 NAC genes that aid in Al^3+^ stress response (Jin et al., 2020). Similar to this study, most predominant TF families identified during transcriptomic analysis of Al^3+^ stress in roots of *Arabidopsis* were MYB, bHLH, and AP2/EREBP (Kumari et al., 2008).

Furthermore, Al^3+^ sequestration within vacuoles by way of ABC transporters was prominently seen in the case of the cultivars. On the other hand, epigenetic regulation by way of chromatin remodelling and histone modifications *via* methyl transferases specifically regulated Al^3+^ stress tolerance in the wild genotype. Lei et al. (2017) have reported that two half-size ABC transporters, *viz*., FeALS1.1 and FeALS1.2, sequestered Al^3+^ into the vacuoles of root and leaf cells, respectively, and thereby helped in internal Al^3+^ detoxification in buckwheat. Similar to the observation of the authors, studies exploring the relationship between epigenetic regulation and Al^3+^ tolerance has evidenced that Al^3+^ tolerance is conferred through DNA methylation (Gallo-Franco et al., 2020), e.g., changes in methylation patterns of genes encoding glycerophosphodiestrase-like proteins have induced Al^3+^ stress in tobacco (Choi and Sano, 2007). Using methylation-sensitive amplification polymorphism, Bednarek et al. (2017) showed that Al^3+^ exposure induced demethylation. Contrary to this, Taspinar et al. (2018) found DNA hypermethylation as a protective response against Al^3+^ stress. The data generated in this analysis can be used in the improvement of the lentil reference genome (knowpulse.usask.ca). Development of understanding of Al tolerance regulation in lentils will expedite lentil breeding programs for improved Al resistance.

## Conclusion

Physio-biochemical, cyto-morphological, and transcriptomic evaluations of Al^3+^ tolerance in lentil have revealed that Al^3+^ stress-responsive pathways are involved in important cellular and biological processes associated with organic acids exudation, callose accumulation, reactive oxygen species detoxification, and phytohormone signalling. The study has elaborated the importance of genes associated with the cell wall and secondary metabolism in providing tolerance towards Al stress. Also, the activation of type I and type II metacaspases in tolerant genotypes has illuminated the role of programmed cell death under Al toxic conditions. In addition to these pathways, the inter- and intraspecies comparison revealed that Al^3+^ sequestration inside vacuoles was predominant in cultivars, whereas epigenetic modifications enhanced Al^3+^ resilience in wild. Comprehensive understanding of metabolic pathways and key genes found to be actively involved in stress signalling, as revealed in this study, will further help in reinforcing lentil breeding programs for enhanced Al^3+^ tolerance.

## Data Availability Statement

The datasets presented in this study can be found in online repositories. The names of the repository/repositories and accession number(s) can be found here: NCBI (SAMN08211543); https://www.ncbi.nlm.nih.gov/biosample/SAMN08211543.

## Author Contributions

DhS, MP, MS, RS, SC, and AS formulated the experiments. DhS, CS, JT, PC, RT, NK, and DeS executed molecular and statistical data analysis. DhS, JT, and CS drafted the manuscript. All the authors have read and approved the final manuscript.

## Conflict of Interest

The authors declare that the research was conducted in the absence of any commercial or financial relationships that could be construed as a potential conflict of interest.

## Publisher's Note

All claims expressed in this article are solely those of the authors and do not necessarily represent those of their affiliated organizations, or those of the publisher, the editors and the reviewers. Any product that may be evaluated in this article, or claim that may be made by its manufacturer, is not guaranteed or endorsed by the publisher.

## Supplementary Material

Supplementary Figure 1 Basic local alignment search tool hits of overall contigs obtained after Al treatment (148 μM AlCl_3_·H_2_O) in lentil genotypes using: (A) species and (B) database.

Supplementary Figure 2 Web gene ontology annotation plot representing the number of genes involved in cellular, molecular, and biological functions under Al stress conditions in combinations 1T-2T, 1T-3T, and 2T-3T, where 1T: L-4602 treated, 2T: BM-4 treated, and 3T: ILWL-15 treated.

Supplementary Table 1 Details of primers used for the validation of genes differentially expressed under Al-stress conditions in lentil through quantitative real-time polymerase chain reaction.

Supplementary Table 2 Significantly up- and down-regulated differentially expressed genes (DEGs) under Al-stress conditions in lentil for different combinations using EdgeR.

Supplementary Table 3 EdgeR data showing the top 20 up-regulated DEGs under Al-stress conditions in lentil for the combination tolerant-sensitive treated.

Supplementary Table 4 EdgeR data showing the top 20 downregulated DEGs under Al-stress conditions in lentil for the combination tolerant treated vs. sensitive treated.

Supplementary Table 5 List of total number of reads, simple sequence repeats, and single nucleotide polymorphisms generated in different lentil samples under Al-stress conditions.

## Abbreviations

Al: aluminium
BLAST: basic local alignment search tool
COG: cluster of orthologous groups
DEGs: differentially expressed genes
FAO: Food and Agriculture Organization
FDR: false discovery rate
GATK: genome analysis tool kit
GO: gene ontology
ICARDA: International Centre for Agricultural Research in Dry Areas
ILWL: ICARDA International Lentil Wild Lines
KEGG: Kyoto Encyclopedia of Genes and Genomes
MISA: Samtoolsmpileup and MIcroSAtellite identification
NBPGR: National Bureau of Plant Genetic Resources
NCBI: National Center for Biotechnology Information non-redundant (nr)
NPF: National Phytotron Facility
PDB: Protein Database Bank
qRT-PCR: quantitative real-time PCR
RefSeq: reference sequence
RNA: ribonucleic acid
ROS: reactive oxygen species
SNPs: single nucleotide polymorphisms
SSRs: simple sequence repeats
SWISSPROT: Swiss-Prot
TAIR: The *Arabidopsis* Information Resource
TF: transcription factor
UNIREF: UniProt Reference
UNIREF: UniProt Reference Clusters
WEGO: web gene ontology annotation.
