# Supplementary figures and images for: Comparative Inter- and IntraSpecies Transcriptomics Revealed Key Differential Pathways Associated With Aluminium Stress Tolerance in Lentil
Source: Front Plant Sci. 2021 Aug 31;12:693630. doi: 10.3389/fpls.2021.693630 (PMC8438445; doi:10.3389/fpls.2021.693630)

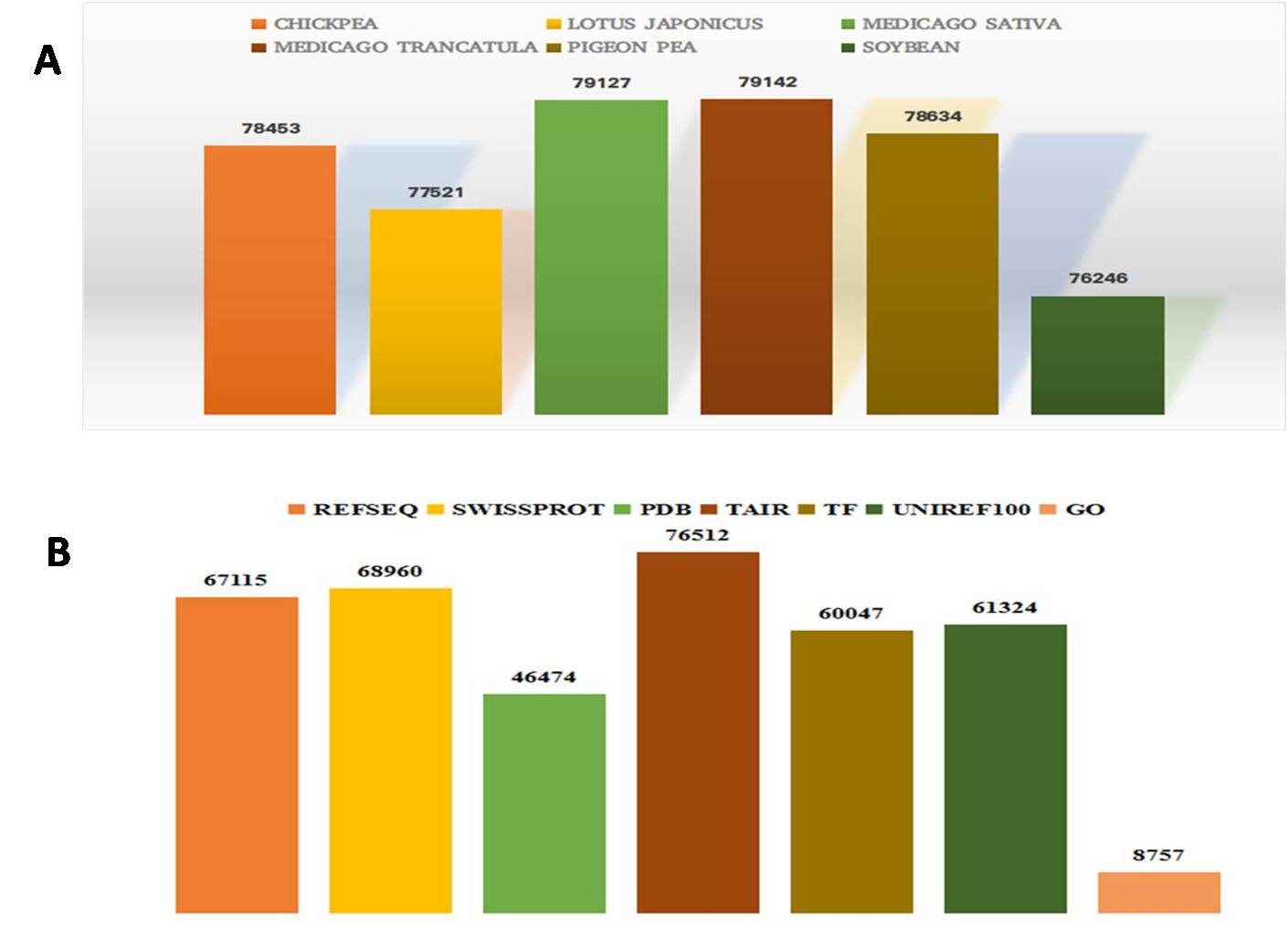

Supplement: Supplementary Figure 1 — Basic local alignment search tool hits of overall contigs obtained after Al treatment (148 μM AlCl3·H2O) in lentil genotypes using: (A) species and (B) database. [file Image_1.JPEG]

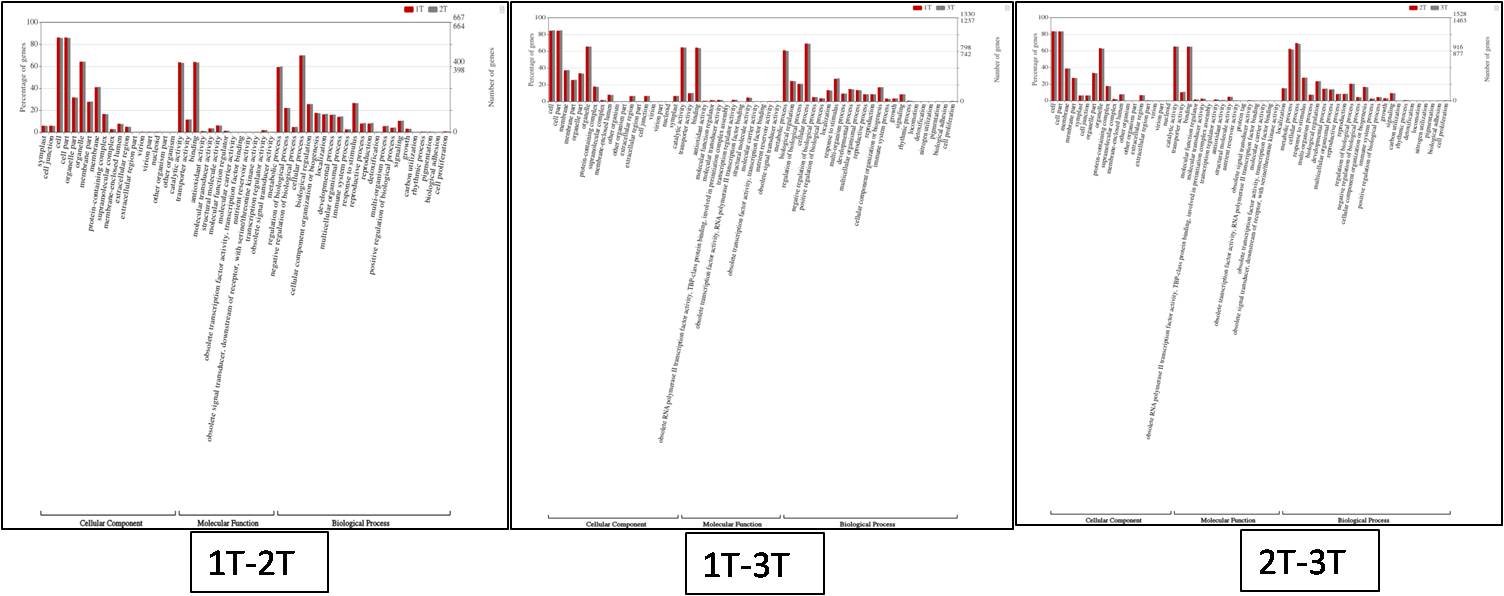

Supplement: Supplementary Figure 2 — Web gene ontology annotation plot representing the number of genes involved in cellular, molecular, and biological functions under Al stress conditions in combinations 1T-2T, 1T-3T, and 2T-3T, where 1T: L-4602 treated, 2T: BM-4 treated, and 3T: ILWL-15 treated. [file Image_2.JPEG]
