# Supplementary material for: Comparative Inter- and IntraSpecies Transcriptomics Revealed Key Differential Pathways Associated With Aluminium Stress Tolerance in Lentil
Source: Front Plant Sci. 2021 Aug 31;12:693630. doi: 10.3389/fpls.2021.693630 (PMC8438445; doi:10.3389/fpls.2021.693630)
Supplement: Supplementary Table 1 — Details of primers used for the validation of genes differentially expressed under Al-stress conditions in lentil through quantitative real-time polymerase chain reaction. [file Table_1.DOC]

Additional file 1.Table S1. List of 12 primers used for validation of NGS data

| **ID** | **PRIMER** | **PRODUCT SIZE** | **FPKM_1C** | **FPKM_1T** | **foldChange** | **log2FoldChange** | **pval** | **DescriptionSP** |
| --- | --- | --- | --- | --- | --- | --- | --- | --- |
| **1C-1T** | | | | | | | | |
| DN49785_c0_g1_i1 | Forward primer TGATCGGTTGCACCAAGTCC Reverse primer GGCCTCCTCCAACTGCATAC | 95bp | 56.06 | 0.95 | 0.01 | -5.87 | 9.26E-43 | Cellulose synthase A catalytic subunit 8 UDP-forming |
| DN82349_c0_g1_i1 | Forward primer ATGTGAAGCCTTTCAGGGCA Reverse primer TCGAAATGTGTTCGGTCGGA | 181bp | 22.28 | 0.61 | 0.02 | -5.18 | 1.52E-16 | Expansin-B3 |
| DN104348_c0_g1_i1 | Forward primer CAAAGTTTGTTGCACACCCAT Reverse primer ACATGGAAGCCTAATTGGACC | 95bp | 1.047 | 73.43 | 70.09 | 6.13 | 2.90E-54 | Auxin-responsive protein SAUR72 |
| DN81353_c1_g2_i1 | Forward primer CGTGATGCTCTTTTCAAGGCAA Reverse primer AAGACAGCAAGAAACCTCGC | 119bp | 4.88 | 462.76 | 94.80 | 6.56 | 0 | ABA-responsive protein ABR18 |
| **2C-2T** | | | | | | | | |
| DN73628_c0_g2_i2 | Forward primer AGTGTTTAAGCCAACAGCCG Reverse primer CAAACCAATCTGGCGCGAAC | 92bp | 22.45 | 0.62 | 0.027 | -5.16 | 1.87E-14 | Peroxidase 15 |
| DN62821_c0_g1_i1 | Forward primer ATTGTCGTTGAATGGAGCGT Reverse primer TACACCGACCACATCGACCT | 112bp | 17.31 | 0.59 | 0.034 | -4.86 | 2.78E-11 | PLASMODESMATA CALLOSE-BINDING PROTEIN 3 |
| DN86113_c0_g2_i1 | Forward primer GGTCTGCGTCGTATTGGTCA Reverse primer AGCCGATACCACGACCTAGA | 181bp | 0.679 | 61.73 | 91.526 | 6.51 | 1.18E-25 | 30S ribosomal protein S5 |
| DN83068_c0_g1_i1 | Forward primer GGCACGGTTTTATCGCACAA Reverse primer CTGAACGAAGTGCAGCAACC | 111bp | 0.71 | 70.68 | 99.501 | 6.63 | 5.60E-27 | Isocitrate lyase |
| **3C-3T** | | | | | | | | |
| DN22208_c0_g1_i1 | Forward primer GGTTTTGGAGGCTCAAGCAAA Reverse primer CTCACACGTCTCTGAATTCCAT | 108bp | 29.38 | 3.40 | 0.11 | -3.10 | 2.17E-10 | Transcription factor UPBEAT1 |
| DN80931_c0_g2_i1 | Forward primer TGTACATGGCACGAGCATAGA Reverse primer GGGACCCTGAAAGATGGGGT | 171bp | 25.08 | 3.08 | 0.12 | -3.02 | 8.53E-09 | Probable beta-1 4-xylosyltransferase IRX9 |
| DN93071_c0_g1_i1 | Forward primer CTTTGCGTCGCGAACGTATG Reverse primer TCCCCACTGTTCAACACTCTC | 102bp | 3.52 | 127.75 | 36.19 | 5.17 | 1.10E-59 | 17.1 kDa class II heat shock protein |
| DN97372_c0_g1_i1 | Forward primer TACCCATGCATCTCCGCTTG Reverse primer CTGAGTTATGGGCTGACCCG | 76bp | 3.52 | 305.20 | 86.46 | 6.43 | 6.42E-156 | Cytochrome P450 81E8 |
