# Supplementary material for: Comparative Inter- and IntraSpecies Transcriptomics Revealed Key Differential Pathways Associated With Aluminium Stress Tolerance in Lentil
Source: Front Plant Sci. 2021 Aug 31;12:693630. doi: 10.3389/fpls.2021.693630 (PMC8438445; doi:10.3389/fpls.2021.693630)
Supplement: Supplementary Table 2 — Significantly up- and down-regulated differentially expressed genes (DEGs) under Al-stress conditions in lentil for different combinations using EdgeR. [file Table_2.DOC]

Table S2. Significantly up and downregulated DEGs for different combinations using EdgeR.

| S. No. | Combination | Total Significant DEGs | Significantly Upregulated | | | Significantly Downregulated | | |
| --- | --- | --- | --- | --- | --- | --- | --- | --- |
|  |  |  | Total | Log_2_FC>1.5 | Log_2_FC = inf. | Total | ABS_Log_2_FC>1.5 | ABS_Log_2_FC= inf. |
| 1 | 1C-1T | 14127 | 7196 | 2329 | 0 | 6931 | 1609 | 0 |
| 2 | 2C-2T | 14312 | 7304 | 2380 | 0 | 7008 | 1562 | 0 |
| 3 | 3C-3T | 18808 | 10624 | 5130 | 0 | 8184 | 2420 | 0 |
| 4 | 1T-2T | 2547 | 1260 | 432 | 0 | 1287 | 368 | 0 |
| 5 | 1T-3T | 12177 | 5645 | 1694 | 0 | 6532 | 2035 | 0 |
| 6 | 2T-3T | 15434 | 7486 | 2430 | 0 | 7948 | 2541 | 0 |
