# Supplementary material for: Comparative Inter- and IntraSpecies Transcriptomics Revealed Key Differential Pathways Associated With Aluminium Stress Tolerance in Lentil
Source: Front Plant Sci. 2021 Aug 31;12:693630. doi: 10.3389/fpls.2021.693630 (PMC8438445; doi:10.3389/fpls.2021.693630)
Supplement: Supplementary Table 3 — EdgeR data showing the top 20 up-regulated DEGs under Al-stress conditions in lentil for the combination tolerant-sensitive treated. [file Table_3.DOC]

Table S3. EdgeR data showing top 20 up-regulated DEGs for the combination tolerant sensitive treated.

| **ID** | **logFC** | **logCPM** | **PValue** | **FDR** | **Description** |
| --- | --- | --- | --- | --- | --- |
| TRINITY_DN54283_c0_g1_i1 | 6.41 | -0.040 | 4.66E-10 | 1.98E-06 | - |
| TRINITY_DN74494_c0_g1_i1 | 6.23 | -0.17 | 7.45E-09 | 1.19E-05 | - |
| TRINITY_DN54609_c0_g1_i1 | 6.18 | -0.20 | 1.49E-08 | 2.03E-05 | - |
| TRINITY_DN10923_c0_g1_i1 | 6.13 | -0.24 | 2.98E-08 | 3.16E-05 | - |
| TRINITY_DN80888_c1_g3_i2 | 6.02 | -0.32 | 1.19E-07 | 7.35E-05 | - |
| TRINITY_DN67301_c0_g1_i1 | 5.90 | -0.40 | 4.77E-07 | 0.000172 | - |
| TRINITY_DN79682_c1_g3_i1 | 5.90 | -0.40 | 4.77E-07 | 0.000172 | Ribosomal RNA large subunit methyltransferase I |
| TRINITY_DN85861_c2_g4_i1 | 5.77 | -0.48 | 1.91E-06 | 0.000476 | PX domain-containing protein EREX |
| TRINITY_DN69746_c0_g1_i1 | 5.63 | -0.57 | 7.63E-06 | 0.001103 | Protein LIGHT-DEPENDENT SHORT HYPOCOTYLS 1 |
| TRINITY_DN73769_c0_g1_i1 | 5.55 | -0.62 | 1.53E-05 | 0.001686 | - |
| TRINITY_DN84834_c0_g1_i1 | 5.47 | -0.67 | 3.05E-05 | 0.002529 | BTB/POZ domain-containing protein NPY1 |
| TRINITY_DN83215_c4_g3_i1 | 5.47 | -0.67 | 3.05E-05 | 0.002529 | - |
| TRINITY_DN79925_c10_g3_i1 | 5.391 | -0.72 | 3.05E-05 | 0.002529 | ATP-dependent zinc metalloprotease FTSH 7 chloroplastic |
| TRINITY_DN73276_c1_g2_i1 | 5.39 | -0.72 | 6.1E-05 | 0.003876 | Transcription factor VOZ1 |
| TRINITY_DN85829_c1_g7_i8 | 5.39 | -0.72 | 6.1E-05 | 0.003876 | - |
| TRINITY_DN5093_c0_g1_i1 | 5.20 | -0.82 | 0.000122 | 0.005959 | - |
| TRINITY_DN61154_c0_g1_i1 | 5.20 | -0.82 | 0.000122 | 0.005959 | - |
| TRINITY_DN70577_c0_g1_i1 | 5.20 | -0.82 | 0.000122 | 0.005959 | - |
| TRINITY_DN84227_c1_g1_i1 | 5.20 | -0.82 | 0.000122 | 0.005959 | - |
| TRINITY_DN105029_c0_g1_i1 | 5.10 | -0.88 | 0.000244 | 0.009523 | F-box protein At5g51380 |
