# Supplementary material for: Comparative Inter- and IntraSpecies Transcriptomics Revealed Key Differential Pathways Associated With Aluminium Stress Tolerance in Lentil
Source: Front Plant Sci. 2021 Aug 31;12:693630. doi: 10.3389/fpls.2021.693630 (PMC8438445; doi:10.3389/fpls.2021.693630)
Supplement: Supplementary Table 4 — EdgeR data showing the top 20 downregulated DEGs under Al-stress conditions in lentil for the combination tolerant treated vs. sensitive treated. [file Table_4.DOC]

Table S4. EdgeR data showing top 20 downregulated DEGs for the combination tolerant treated v/s sensitive treated.

| **ID** | **logFC** | **logCPM** | **PValue** | **FDR** | **Description SP** |
| --- | --- | --- | --- | --- | --- |
| TRINITY_DN81385_c0_g1_i1 | -6.08 | -0.34 | 5.96E-08 | 4.71E-05 | Zinc-finger homeodomain protein 4 |
| TRINITY_DN79375_c1_g1_i1 | -5.95 | -0.42 | 2.38E-07 | 0.000121 | - |
| TRINITY_DN37649_c0_g1_i1 | -5.82 | -0.51 | 9.54E-07 | 0.000289 | - |
| TRINITY_DN58295_c0_g1_i1 | -5.82 | -0.51 | 9.54E-07 | 0.000289 | - |
| TRINITY_DN19151_c0_g1_i1 | -5.67 | -0.60 | 3.81E-06 | 0.000712 | - |
| TRINITY_DN65023_c1_g2_i1 | -5.67 | -0.60 | 3.81E-06 | 0.000712 | - |
| TRINITY_DN65534_c0_g1_i1 | -5.67 | -0.60 | 3.81E-06 | 0.000712 | - |
| TRINITY_DN61981_c0_g1_i2 | -5.59 | -0.65 | 1.53E-05 | 0.001686 | - |
| TRINITY_DN80647_c0_g2_i1 | -5.59 | -0.65 | 1.53E-05 | 0.001686 | Probable carboxylesterase 11 |
| TRINITY_DN23470_c0_g1_i1 | -5.50 | -0.70 | 3.05E-05 | 0.002529 | Peptidyl-prolyl cis-trans isomerase 1 |
| TRINITY_DN81336_c0_g2_i2 | -5.50 | -0.70 | 3.05E-05 | 0.002529 | Aldehyde dehydrogenase family 3 member F1 |
| TRINITY_DN64108_c1_g1_i1 | -5.41 | -0.76 | 6.1E-05 | 0.003876 | - |
| TRINITY_DN64247_c0_g1_i1 | -5.41 | -0.76 | 6.1E-05 | 0.003876 | G-type lectin S-receptor-like serine/threonine-protein kinase SD1-13 |
| TRINITY_DN69251_c0_g1_i1 | -5.41 | -0.76 | 6.1E-05 | 0.003876 | Putative lysine-specific demethylase JMJ16 |
| TRINITY_DN84985_c7_g4_i1 | -5.41 | -0.76 | 6.1E-05 | 0.003876 | ABC transporter B family member 6 |
| TRINITY_DN77161_c0_g1_i2 | -5.41 | -0.76 | 6.1E-05 | 0.003876 | Protein PLANT CADMIUM RESISTANCE 8 |
| TRINITY_DN50496_c0_g1_i1 | -5.31 | -0.81 | 0.000122 | 0.005959 | - |
| TRINITY_DN84809_c0_g1_i1 | -5.31 | -0.81 | 0.000122 | 0.005959 | - |
| TRINITY_DN85519_c2_g2_i1 | -5.31 | -0.81 | 0.000122 | 0.005959 | - |
| TRINITY_DN50613_c0_g1_i1 | -5.21533 | -0.87416 | 0.000244 | 0.009523 | Peptidyl serine alpha-galactosyltransferase |
